# Modeling Music-Selection Behavior in Everyday Life: A Multilevel Statistical Learning Approach and Mediation Analysis of Experience Sampling Data

**DOI:** 10.3389/fpsyg.2019.00390

**Published:** 2019-03-19

**Authors:** Fabian Greb, Jochen Steffens, Wolff Schlotz

**Affiliations:** ^1^Max Planck Institute for Empirical Aesthetics, Frankfurt am Main, Germany; ^2^Audio Communication Group, Technische Universität Berlin, Berlin, Germany; ^3^Institute of Psychology, Goethe University, Frankfurt am Main, Germany

**Keywords:** music-listening behavior, music-selection behavior, functions of music listening, machine learning, experience sampling method, user behavior analysis

## Abstract

Music listening has become a highly individualized activity with smartphones and music streaming services providing listeners with absolute freedom to listen to any kind of music in any situation. Until now, little has been written about the processes underlying the selection of music in daily life. The present study aimed to disentangle some of the complex processes among the listener, situation, and functions of music listening involved in music selection. Utilizing the experience sampling method, data were collected from 119 participants using a smartphone application. For 10 consecutive days, participants received 14 prompts using stratified-random sampling throughout the day and reported on their music-listening behavior. Statistical learning procedures on multilevel regression models and multilevel structural equation modeling were used to determine the most important predictors and analyze mediation processes between person, situation, functions of listening, and music selection. Results revealed that the features of music selected in daily life were predominantly determined by situational characteristics, whereas consistent individual differences were of minor importance. Functions of music listening were found to act as a mediator between characteristics of the situation and music-selection behavior. We further observed several significant random effects, which indicated that individuals differed in how situational variables affected their music selection behavior. Our findings suggest a need to shift the focus of music-listening research from individual differences to situational influences, including potential person-situation interactions.

## Introduction

Music listening in recent years has become a highly individualized activity. The rapid growth of music digitalization and mobile music listening devices, such as smartphones and music streaming services, provide individuals with the freedom to listen to almost any kind of music during their daily life (Berthelmann, 2017; Gesellschaft für Konsumforschung, 2017). Given this freedom of choice, people indeed tend to actively select and use music to accomplish specific goals in certain situations (DeNora, 2000; Krause et al., 2015). In contrast to the widespread use of new technological developments by music listeners, little is known about the processes underlying the selection of music in daily life, and scientific research about music listening in everyday life still is underdeveloped. To some extent, the high degree of complexity due to the large amount of contributing factors and their interactions has led to this lack of current knowledge. Thus, the goal of the current study was to explain this complexity when people actively select music in their daily life. In particular, we aimed to identify personal and situational variables of high relevance for music-selection behavior, and to integrate these factors into a comprehensive model predicting music selection while strictly avoiding overfitting. This also includes an investigation of the role that functions of music listening play in the selection of music. By using the experience sampling method, we captured almost unbiased behavioral data representative of participants' daily lives. We used statistical learning procedures for variable selection to make predictions of unseen data and avoid overfitting (Yarkoni and Westfall, 2017). Clarifying the role of listener, situation, and functions of music listening in music selection aids in the understanding of why people listen to music in certain situations and how situational and person-related factors govern the selection of music and its characteristics. This knowledge helps to answer the question of who listens to what kind of music in which situation and why, and might contribute to an improvement of music recommendation systems.

### Contributions of Person and Situation to Music-Listening Behavior

Past research on music listening mostly focused on one of two major determinants of music-listening behavior, namely influences of individual or situational factors. Research on individual differences mainly seeks to answer questions such as why some people predominantly listen to aggressive rock music, whereas others prefer listening to smooth jazz (Delsing et al., 2008; Gardikiotis and Baltzis, 2012) or why some individuals mainly listen to music for intellectual stimulation and others use it for mood regulation (e.g., Chamorro-Premuzic and Furnham, 2007). This research revealed a large number of significant associations between music listening and person-related variables, particularly age, gender, personality traits, musical taste, and musical training (e.g., LeBlanc et al., 1999; Chamorro-Premuzic et al., 2009; Boer et al., 2012; Ferwerda et al., 2015; Greenberg et al., 2015; Cohrdes et al., 2017).

Complementary research investigating situational influences on music listening addresses questions such as where people listen to music (e.g., Sloboda et al., 2001; North et al., 2004), why people listen to music in everyday life (e.g., North et al., 2004; Randall and Rickard, 2017b), with whom they listen to music (e.g., North et al., 2004; Liljestrom et al., 2013), when they listen to music (North et al., 2004; Krause et al., 2016), during which activity people listen to music (North et al., 2004; Juslin et al., 2008), and how these situational factors influence the selection, judgement, or experience of the music (North et al., 2004; Juslin et al., 2008; Greasley and Lamont, 2011; Krause et al., 2016; Krause and North, 2017a,b; Randall and Rickard, 2017b).

Since both person and situation usually influence behavior at the same time, investigating variables of both domains simultaneously is of high importance. Combining this synthesis approach proposed by Fleeson and Noftle (2008) with daily-life research methods (Mehl and Conner, 2012) can potentially provide more reliable results as well as more valid conclusions and behavioral predictions. Integrating both levels of variance better reflects the complexity of the multitude of factors interacting in daily life. The simultaneous investigation of individual and situational influences also allows an estimate of the amount of variance explained by both domains. In music psychology, research integrating person-related and situational factors is scarce. The few existing studies indicated that both domains are important for explaining the presence of music (Krause and North, 2017b), emotional responses to music (Randall and Rickard, 2017a), or functions of music listening in different situations (Greb et al., 2018a). Up to now, only one study specifically addressed music-selection behavior (Greb et al., 2018b). This study showed that the characteristics of selected music are largely attributable to situational influences and it revealed a detailed pattern of variables being associated with the selection of music (Greb et al., 2018b). Functions of music listening referring to the intentional use of music to accomplish specific goals were the most important variables for predicting music selection (Greb et al., 2018b). However, the study relied on retrospective self-reports of three listening situations obtained via an online survey, potentially introducing bias to the data.

Functions of music listening also vary by situation (North et al., 2004) and are largely influenced by the activity performed while listening to music (Greb et al., 2018a). In addition, functions were shown to reliably predict music selection in specific situations (Randall and Rickard, 2017a; Greb et al., 2018b). Hence, functions of music listening might mediate music selection in daily life, such that activity or mood determines why a person wants to listen to music in a given situation, whereas the subsequent process of selecting a specific musical piece is largely driven by these functions of music listening. Thus, the specific role of musical functions in the process of music selection needs further clarification.

### Methodological Challenges

Investigating music-selection behavior in daily life is associated with considerable methodological challenges. First, measuring real-life behavior requires a suitable data collection method. Many of the studies mentioned above used retrospective data collection based on online surveys or laboratory studies, which are relatively easy to conduct but are limited in their ecological validity and are likely to be biased in several ways (e.g., memory biases, limited representativeness of situations). The gold standard of investigating real-life behavior in ecologically valid settings leading to almost unbiased data is the collection of data in people's daily life. To measure subjective perceptions and experiences involved during music listening and selection, the experience sampling method (ESM) was identified as a suitable method (Sloboda et al., 2001; Hektner et al., 2007; Greasley and Lamont, 2011; Randall and Rickard, 2013). ESM provides a multitude of data points per participant that allows the investigation of between- (i.e., person-related) and within-subject (i.e., situational) variance (Hektner et al., 2007). The widespread distribution of smartphones makes it easier to conduct ESM studies compared with the past when people had to carry around large extra devices (e.g., palmtop computers).

Second, a data collection method investigating person-related and situational factors simultaneously requires appropriate statistical models. Multilevel modeling (MLM) is the most appropriate method for analyzing nested or longitudinal data, as it allows modeling of several levels of variance simultaneously and estimation of the relative impact of person-related and situational factors on the outcome variable (Nezlek, 2008). ESM data with its nested structure in combination with MLM can be used for building reliable models to predict real-life behavior (Fleeson, 2007). In music psychology, this possibility has often been neglected and ESM data were averaged at the listener level while ignoring situational variance (e.g., Juslin et al., 2008; Greasley and Lamont, 2011).

Third, the proposed approach of investigating person-related and situational factors in an integrative model inevitably leads to a large number of variables to be included in the analysis (e.g., Randall and Rickard, 2017a; Greb et al., 2018b). Consequently, the question of which variables should be selected as the most significant predictors of behavior becomes an important issue. Commonly used selection procedures, such as all sorts of step-wise regression, are highly problematic as they often lead to overestimation of regression coefficients and tend to select irrelevant predictors (Derksen and Keselman, 1992; Steyerberg et al., 1999; Whittingham et al., 2006; Flom and Cassell, 2007). These problems—also known as overfitting—are addressed by the field of statistical learning, which has developed a broad set of methods and procedures to overcome such limitations (Babyak, 2004; Chapman et al., 2016). Many of these methods provide new opportunities to enrich psychological research (Chapman et al., 2016; Yarkoni and Westfall, 2017). For instance, the least absolute shrinkage and selection operator (Lasso), originally proposed by Tibshirani (1996), offers a promising alternative to common variable selection procedures. As the Lasso is applicable on linear regression and multilevel linear regression models, it is especially useful if researchers aim to interpret model coefficients, as is often the case in psychological research. Especially, the percentile-Lasso—an empirical extension of the original Lasso—was shown to select very few false positives (i.e., irrelevant predictors) by using repeated cross-validation and thereby focusing on prediction (Roberts and Nowak, 2014). In addition, it features a penalization term that shrinks the coefficients toward smaller values and thereby avoids overestimation of coefficients. Notably, the issue of overfitting and the application of statistical learning to overcome such limitations have rarely found its way into music psychology. In the context of music listening in daily life, only one study has successfully applied cross-validation and a Lasso algorithm for variable selection to find the most significant predictors of music-selection behavior (i.e., Greb et al., 2018b). However, that study employed retrospective assessments and a very limited variety of situations, which might facilitate biased results despite appropriate statistical analysis based on statistical learning.

### Present Research

**Figure 1:**
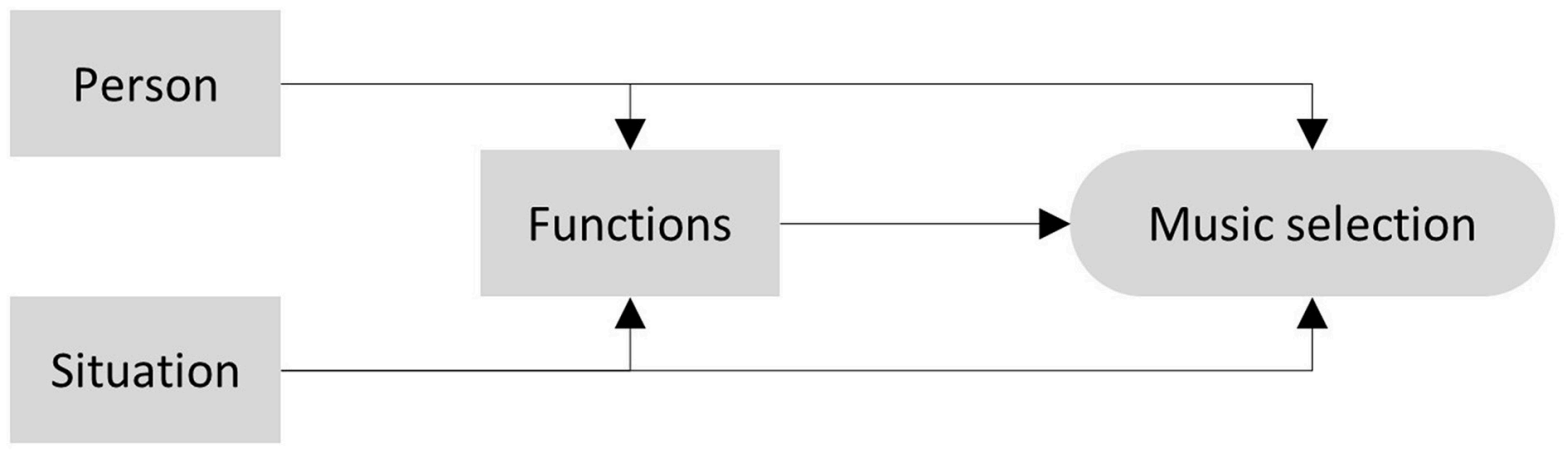
Model of music selection guiding the current investigation.

We sought to explore music-selection behavior in daily life by investigating situational and person-related factors simultaneously. Given the research findings and theoretical considerations above, our research was guided by the model shown in Figure 1. To take the multitude of potentially influential factors in all domains (person, situation, functions) into account simultaneously, we built comprehensive models by including a broad set of variables. With the greater objectives of avoiding overfitting and maximizing predictive accuracy, our study had the following research aims:

1. Investigate the relative contribution of person-related and situational variables to variance in daily-life music-selection behavior (i.e., estimating between- and within-subject variance components).
2. Identify the most important variables involved in the process of music selection as outlined in Figure 1 (i.e., detect all relevant direct effects).
3. Identify the potential mediating role of functions of music listening in the association of situational and person-related variables with music selection.
4. Explore whether effects of situational variables on music selection vary across individuals by testing for individual differences in the associations identified earlier (i.e., effects resulting from research aim 2).
5. As we consider replication to be an important aspect of our research, we aimed at comparing the results of the current study using daily-life research methodology to those of another study that used the same statistical approach but was based on retrospective reports of very few music listening situations (i.e., Greb et al., 2018b).

To address these aims, we conducted an experience sampling study in which participants reported on their music listening using smartphones. Participants reported on situational cues, the music they heard, and on functions of music listening. In addition, we collected a broad set of person-related variables in an initial laboratory session. Our study design is an improvement of a previous study in which we investigated all direct effects on music selection in daily life (Greb et al., 2018b). To address the methodological problems discussed in the introduction and to be able to compare results, we applied the same statistical learning procedure (i.e., percentile-Lasso) as that study.

## Method

All experimental procedures were ethically approved by the Ethics Council of the Max Planck Society, and were undertaken with written informed consent of each participant.

### Sample

In total, 119 participants (54 men, 65 women; mean = 24.4 years; *SD* = 4.4) were recruited via the participant database of the Max Planck Institute for Empirical Aesthetics. To ensure sufficient within-subject variance, we only included participants who indicated listening to music for at least 2 h a day for a minimum of 5 days per week. People received 25 € for voluntarily participating in the study. Depending on the amount of valid responses to prompts, each participant could receive a graded bonus of up to 25 € (for details see the procedure section).

### Measures

#### Prescreening

Frequency of music listening in daily life was measured by two items: (1) “How often do you listen to music during the week?” (response scale with nine scale points: less than once a week, once, twice, thrice, four times, five times, six times, seven times, more than seven times a week); and (2) “On average, how long do you listen to music per day?” (response scale with nine scale points: <0.5, 0.5, 1, 1.5, 2, 2.5, 3, 4 h, more than 4 h). Additionally, we asked participants to report if they owned a smartphone and, if yes, which operating system is running on their device (Android, iOS, Windows Mobile, Blackberry, other).

#### Person-Related Variables

In addition to age and gender, we assessed musical sophistication using the German version of the Gold-MSI (Schaal et al., 2014), the intensity of music preference using six items from Schäfer and Sedlmeier (2009), musical taste using liking ratings for 19 musical styles (see Greb et al., 2018a for details), and the Big Five personality traits using a German version of the IPIP-NEO-120 (Johnson, 2014) translated by Treiber et al. (2013). For the musical taste ratings, we computed sum scores based on the factor structure reported by Greb et al. (2018a). As the questions about musical taste included the possibility to select “I don't know” for a musical style, we used imputation to replace missing data with the mean value of the ratings of the respective musical style.

#### ESM Measures

Each assessment started with the initial question “Are you listening to music right now?” If the answer was “no,” the assessment was finished; if the answer was “yes,” it continued. The remainder of the assessment consisted of three sections about the situation, the music, and the functions of music listening in the current situation. The first section asked participants to indicate how long they have been listening to music already, what their main activity was using a list of categories developed by Greb et al. (2018a), if other people were present, if they chose the music, and how much control they had in what music they were listening to (see Supplementary Material online for exact wording and response scales). Additionally, we asked for their mood at the time they decided to listen to music [valence and arousal (Russell, 1980)]. We also asked how important participants considered their mood state for the decision to listen to music and how much attention they were paying to the music. The second section included questions about musical characteristics as well as the composer/interpreter, name of the piece, and musical style. First, participants reported on the volume (*quiet–loud*) and their liking of the music (*I like it less–I like it a lot*) on seven-step bipolar rating scales. Musical characteristics were measured by seven items from Greb et al. (2018b)—specifically designed to easily describe music in daily life—on bipolar rating scales with seven scale points, but here we added one item (intensity) for completeness, resulting in the following list of items: *calming–exciting, slow–fast, sad–happy, less melodic–very melodic, less rhythmic–very rhythmic, simple–complex, peaceful–aggressive, less intense–very intense*. Additionally, we asked for familiarity of the music (*unfamiliar–familiar*) and asked the participants to differentiate whether they listened to vocal or instrumental music. Furthermore, we requested participants to name the specific piece, the artists, or the musical style they were listening to at the time of measurement. Given the wide range of different styles people might listen to, we used an open-ended response format, as this was shown to suit this kind of questions best (Greasley et al., 2013). The third section about functions of music listening used a subset of functions developed by Greb et al. (2018a). To keep the ESM measures within each assessment as short as possible but as comprehensive as necessary we took 15 items out of the 22 items comprising the questionnaire by Greb et al. (2018a). In detail, we took three items per dimension that showed the highest factor loadings in Greb et al. (2018a). These 15 items should therefore represent the same dimensions as found by Greb et al. (2018a), namely intellectual stimulation, mind wandering & emotional involvement, motor synchronization & enhanced well-being, updating one's musical knowledge, and killing time & overcoming loneliness (all items are listed in the Supplementary Material). As people might use different functions of music listening with different intensities in everyday life, we assessed functions using a seven-step bipolar rating scale (Not at all–Fully agree). For example, a person might attentively listen to music for intellectual stimulation (high intensity) while, at the same time, aims at updating his/her musical knowledge, but to a lesser degree. We computed sum scores based on the factor structure reported by Greb et al. (2018a).

The following variables—being part of another research project—were not part of the current analyses: duration of music listening at time of measurement, familiarity of the music, liking of the music, instrumental/vocal music, and free responses on musical pieces, artists, and styles.

### Sampling Design and Hardware

The prescreening was completed online through Unipark/EFS Survey software (Questback GmbH, 2015). Person-related variables were reported on a tablet computer (Samsung Galaxy Tab A 1.7) in the laboratory. The ESM measures (daily-life assessments) were presented using movisensXS, Version 1.0.1 (movisens GmbH, 2015), a smartphone application for Android specifically programmed for ESM studies. Participants used either their own smartphone or a loan device (Motorola Moto G3) to run the application.

The study ran for 10 consecutive days (Friday–Sunday). Participants each received 14 alarms within an individual 14-h time window per day. The number of alarms was pretested in a pilot study and was considered acceptable by our pretesting candidates. The alarms occurred randomly within the pre-selected period with a minimum time of 20 min between each alarms. Participants were instructed to answer as many alarms as possible, but they could postpone (by 5, 10, or 15 min) or reject alarms. In addition to this strictly time-based sampling plan, we implemented an event-based plan to capture as many music listening situations as possible. Participants were encouraged to start the assessment manually when they were listening to music by pressing a button in the movisensXS application.

### Procedure

Participants received an e-mail containing an individual participation link. After clicking on the link, they were redirected to an online survey and answered the first questionnaire (prescreening). Participants who fulfilled the inclusion criteria for attending in our main study (i.e., reported listening to music on average for a minimum of 2 h a day for at least 5 days a week) could choose a date for their first session in the lab. People who did not meet the inclusion criteria were informed that they could not participate in the study and were thanked for their time. At their first appointment in the lab, participants completed the questionnaire containing the person-related variables. Afterwards they were informed about the general procedure of the ESM study. Participants who owned an Android smartphone were asked if they were willing to use their own smartphone for the study. All others received a loan device with movisensXS as the only usable application installed. Participants who decided to use their own device received free wireless internet access and guidance for downloading and installing movisensXS from the Google Play store. Thereafter, a demo version of the study was transferred and started. Participants were shown how to accept, delay, or reject an alarm and then simulated a situation in which they were listening to music and answered the items. When participants were familiar with the questionnaire and the handling of the application, they were asked to indicate three 14-h periods between 00:01 and 23:59 they were willing to receive alarms. We chose three blocks—Monday–Thursday, Friday & Saturday, and Sunday—as we expected people to get up earlier during workdays and eventually stay up longer on Friday and Saturday. People were free to choose different periods or use the same period for all assessment days. The event-based (button-pressed) assessments could also be activated outside of the individually selected periods. Participants then received details about the reimbursement. To encourage the participants to answer as many alarms as possible, we decided to employ a graded system. People received 25 € for their participation when answering <50% of random alarms. For each additional 1%−10% of answered alarms they received 5 € extra. This led to a maximum compensation of 50 € if 90%−100% of all alarms were answered. Participants were explicitly instructed that any answer—including “no” I do not listen to music—was counted as an answered alarm to avoid false reporting of music-listening situations to receive higher compensation. Event-based (button-pressed) assessments were not considered for the calculation of the reimbursement. Finally, participants received a small booklet that contained information about the study and contact addresses should they encounter problems.

In the final lab session—after the 10 days of experience sampling—the researcher controlled and transferred the data of the movisensXS application. At this time, participants completed a short evaluation questionnaire and received their reimbursement. Finally, participants received assistance with de-installing movisensXS from their smartphone.

### Data Analysis

As the major aim of the study was to predict music-selection behavior (i.e., active selection of music), we excluded all situations in which participants indicated that they did not have any control about the music in a given situation (“How much control do you have in what you hear?” 1 = No control). In addition, we excluded situations in which participants did not choose the music (“Did you choose the music?” “No”) or listened to music at a club or in a concert. The final data included 2,674 situations reported by 119 participants.

Time data was centered at each participant's earliest response to a random trigger depending on weekdays and weekends. As participants were free to report music listening at any time (button-pressed), very few listening events (3%) were reported shortly before an individual's earliest random trigger. We decided to treat button-pressed events in close proximity to a participant's centering time as “getting up earlier,” whereas time stamps earlier than 2 h before an individual's centering time were considered as “still being awake”. For example, when a participant's earliest answer to a random trigger was 7 a.m., an answer at 8:30 was counted as 1.5, an answer at 6:30 as −0.5, and an answer at 4:30 am was counted as 21.5.

The resulting ESM data reflects a three-level structure (i.e., situations nested within days nested within persons). We checked the different levels of variance and decided not to include days as a separate level, as days explained only minor variability in the outcome variables. The resulting two-level model also is less complex and more readily comparable with that reported by Greb et al. (2018b). In addition, the Lasso implemented in the glmmLasso package (Groll, 2017) used here and by Greb et al. (2018b) cannot estimate three-level models.

We used multilevel linear regressions to model our data, as it allows the analysis of unbalanced designs and the inclusion of time-varying (i.e., situation-related) predictors, while accounting for non-independence of observations within participants. All variables that varied at the within-subject level were centered at the person-mean to clearly differentiate levels of variation (Enders and Tofighi, 2007).

As we considered the sampling of situations within participants to result in a good representation of a person's episodes of music listening in daily life, we also took aggregated measures of all situational variables into account. In the case of continuous situational variables such as arousal, the aggregated measure represents a person's average arousal level while listening to music across situations. For categorical situational variables such as presence of others, this aggregated measure was calculated for each category. It reflects the individual average frequency of that category (e.g., “others present & no interaction”) while listening to music across situations. These aggregated measures describe individual differences in the context (mood, presence of others, etc.) of listening to music, and can be used to predict individual differences in music-selection behavior.

We used intercept-only models to estimate the intraclass correlation coefficient (ICC), which indicates variance components of person and situation levels for functions of music listening and music selection.

To identify the most important variables and to explore all direct effects involved in the music selection process as outlined in our model (Figure 1), our analysis consisted of four steps. These steps followed the logic of a classical mediation analysis as proposed by Baron and Kenney (1986), but considered multiple predictors simultaneously. Step A tested all direct effects of person- and situation-related variables on music selection (i.e., y on x), step B tested all relevant direct effects of person- and situation-related variables on functions of music listening (i.e., m on x), and step C tested all direct effects of musical functions on music selection (i.e., y on m). Finally, step D tested all direct effects of person, situation, and musical functions on music selection (i.e., y on x and m). Step D represents a replication of the statistical analysis by Greb et al. (2018b). Throughout these analyses, we implemented the percentile-Lasso method proposed by Roberts and Nowak (2014), using the 95th percentile for variable selection. We repeated 100 5-fold cross validations with a random sample split for each repetition. For each outcome variable, we determined λ_max_ by successively increasing λ by one until all coefficients were set to zero. We then used a linear λ grid of length 100 running from λ_max_ to zero. Data was split into training and test set at the level of the individual (Level 2), such that models were optimized to make predictions on unseen participants (Roberts et al., 2017). The following lines illustrate model equations entered into the percentile-Lasso procedure for Step D, which includes all covariates analyzed here (see Supplementary Material for model equations of steps A, B, and C):

Level 1:

$$\begin{array}{l} Y_{ij}=\;\beta_{0j}+\beta_{1}\;time_{ij}+\beta_{2}\;time_{ij}^{2}+\beta_{3}\;weekend_{ij}\;\\ \;+\beta_{4}\;valence\;C_{ij}+\beta_{5}\;arousal\;C_{ij}+\beta_{6}\;imp.\;of.\;mood\;C_{ij}\\ \;+\beta_{7}\;attention\;C_{ij}+\beta_{8}\;activity\;1C_{ij}+\ldots \\ \;+\beta_{18}\;activity\;11C_{ij}+\beta_{19}\;presence.of.\;others1C_{ij}\\ \;+\beta_{20}\;presence.of.\;others\;2C_{ij}+\beta_{21}\;intel.stimulation\;C_{ij}\\ \;+\beta_{22}\;mind.\;wandering\;C_{ij}+\beta_{23}\;motor.\;synchronization\;C_{ij}\\ \;+\beta_{24}\;updating.\;musical.\;knowledge\;C_{ij}+\beta_{25}\;killing.\;time\;C_{ij}+R_{ij} \end{array}$$

Level 2:

$$\begin{array}{l} \beta_{0j}=\gamma_{00}+\gamma_{01}\;valence\;M_{j}+\gamma_{02}\;arousal\;M_{j}\\ \;+\gamma_{03}\;imp.of.\;mood\;M_{j}+\gamma_{04}\;attention\;M_{j}\\ \;+\gamma_{05}\;activity\;1M_{j}+\ldots +\gamma_{015}\;activity\;11M_{j}\\ \;+\gamma_{016}\;presence.of.\;others\;1M_{j}+\gamma_{017}\;presence.of.others\;2M_{j}\\ \;+\gamma_{018}\;sex_{j}+\gamma_{019}\;age_{j}+\gamma_{020}\;intensity.\;musicpreference_{j}\\ \;+\gamma_{021}\;musical.\;taste1_{j}+\ldots +\gamma_{026}\;musical.\;taste6_{j}\\ \;+\gamma_{027}\;big5.1_{j}+\ldots +\gamma_{031}\;big5.5_{j}+\gamma_{032}\;gold.\;msi1_{j}+\ldots \\ \;+\gamma_{036}\;gold.\;msi5_{j}+\gamma_{037}\;intel.\;stimulationM_{j}\\ \;+\gamma_{038}\;mind.\;wandering\;M_{j}+\gamma_{039}\;motor.\;synchronization\;M_{j}\\ \;+\gamma_{040}updating.musical.knowledgeM_{j}+\gamma_{041}killing.timeM_{j}+U_{0j} \end{array}$$

where *Y*_*ij*_ denotes the expected musical characteristic selected by person *j* at situation *i* and β_0j_ represents a participant-specific intercept. This intercept is modeled following the second equation including all person-related variables. Within-subject effects are represented by the beta coefficients (β_1_-β_25_) and γ_01_-γ_041_ represent between-subject effects. Capital letter C denotes within-subject centered variables and M denotes aggregated variables at the person level. The terms *R*_*ij*_ and *U*_*j*_ denote residuals at Levels 1 and 2.

For the categorical variables “activity” and “presence.of.others,” we used the group Lasso estimator as implemented in the glmmLasso package (Groll, 2017). This group Lasso estimator treats all categories (i.e., dummy variables) of a categorical variable as belonging together and therefore either includes all categories or excludes all categories pertaining to a categorical variable (Yuan and Lin, 2006; Meier et al., 2008; for details see Groll and Tutz, 2014). *P*-values of non-zero coefficients were estimated by Fisher scoring re-estimation as implemented in glmmLasso (Groll, 2017).

To obtain an overview of the holistic mediation analysis, we calculated a consistency indicator *I*_*F*_ including the number of direct associations for each step of analysis. *I*_*F*_ was calculated as

$$I_{F}=\;\frac{\sum_{1}^{i}s_{i}}{m\times i}$$

Where *s*_*i*_ is the amount of direct effects of variable *s* across the *m* models of the respective step (i.e., eight for musical characteristics during step A, B, D and five for functions of music listening during step C) and *i* denotes the number of variables showing direct effects on at least one of the eight outcome variables of step A. For steps B and C, only those variables were considered which already revealed a direct effect in step A (following the logic of a mediation analysis that a direct effect of y on x is mandatory). Given that the percentile-Lasso selects the most important variables, this indicator should decrease if variables selected during step A were not selected during step D. This decrease would indicate the presence of full mediations.

While these steps provided a holistic overview of all variables and effects involved in music selection, they do not directly provide estimates of direct and indirect effects of person- and situation-related variables on music-selection behavior via functions of music listening. Based on the results of the analysis described above, we constructed and tested mediation models for each outcome using multilevel structural equation modeling (MSEM; Preacher et al., 2010). We selected all person- and situation-related variables of steps A and C that showed significant and robust direct effects on music selection as indicated by the percentile-Lasso method. From this set, we then selected those variables that also showed a significant and robust direct effect on the proposed mediating variable (i.e., functions of music listening as indicated by step B). As mentioned earlier, we used a group Lasso estimator that either includes the complete set of dummy variables pertaining to one categorical variable or excludes them all. However, our aim was to keep models as parsimonious as possible. Therefore, we selected single significant dummy variables, but not the full set. Based on these selection criteria, we built one multilevel structural equation model for each of the eight musical characteristics.

To explore individual differences in associations between predictor variables and music-selection behavior, we re-estimated models based on step D using the lme4 package (Bates et al., 2015) and included random slopes for all situational variables that had shown significant associations during the re-estimation step of the glmmLasso package. Significance of these random parameters was tested by likelihood ratio tests using the lmerTest package (Kuznetsova et al., 2015).

MSEM mediation analyses were performed using the software Mplus v.7.3 (Muthén and Muthén, 1998-2017). All other analyses were performed within the development environment R-Studio (R Studio Team, 2015) of the software R.3.0.2 (R Core Team, 2015).

**Figure 2:**
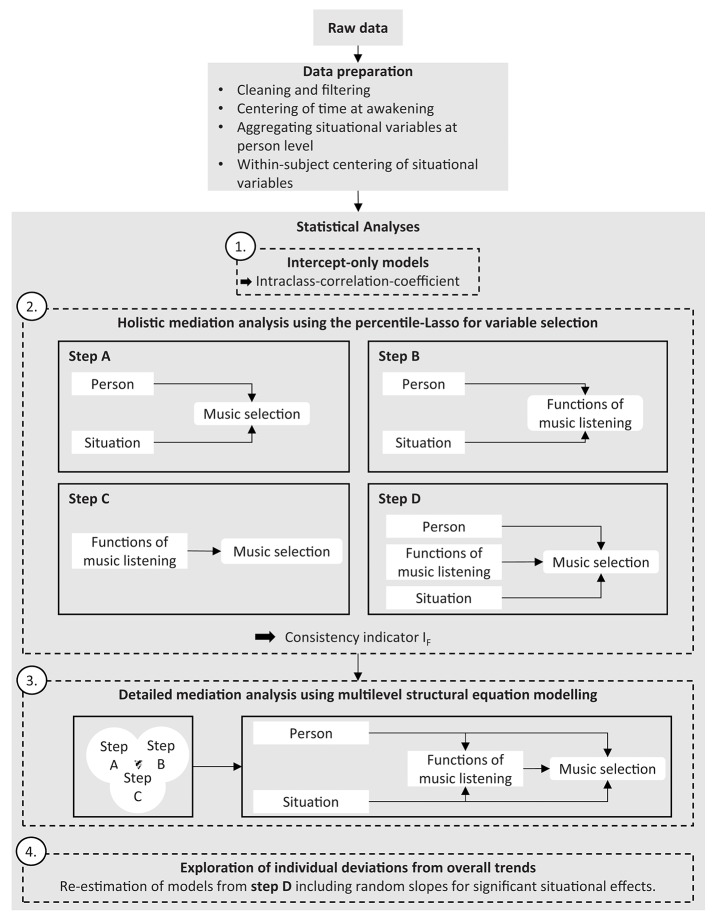
Pipeline detailing major steps from raw data to statistical analysis. For further details about the single steps see the data analysis section.

All major steps of the analyses can be found in Figure 2.

## Results

### Compliance Rate

Of the 15,708 random triggers sent during the study, 117 (0.7%) were dismissed, 2,446 (15.6%) were ignored, and 62 (0.4%) were answered but not finished. This resulted in an overall compliance rate of 83% (13,083 out of 15,708 cases). Participants additionally reported 542 music listening situations by pressing the event button; 23 of those (2.7%) were incomplete.

### Descriptive Statistics

Participants reported 3,564 music-listening situations. In 523 situations, participants did not choose to listen to music; in 25 situations, participants reported listening to music in a concert; in 28 situations, participants were listening to music in a club; and in 676 situations, they reported not having any control over the music. Of the 2,674 music-listening situations considered for the present analysis, 2,202 were based on random triggers and 472 were reported voluntarily by pressing the event button. Participants reported a mean of 3 (*SD* = 2.3, Mdn = 2) listening situations per day, with an average of 7.5 music listening days (*SD* = 2.3), resulting in 22.5 (*SD* = 17.6) listening situations per participant throughout the study. None of the distributions of functions of music listening indicated bimodality, supporting our assumption that functions of music listening in daily life vary in their intensity, and thus are continuous rather than categorical in nature. The following frequencies of main activities while listening to music were reported: being on the move (518; 19%), working & studying (476; 18%), pure music listening (476; 18%), household activity (328; 12%), other activity (230; 9%), social activity (170; 6%), relaxing & falling asleep (147; 5%), personal hygiene (132; 5%), exercise (68; 3%), coping with emotions (50; 2%), making music (50; 2%), and party (17;1%).

### Variance Components

The ICC indicates the relative amount of variance in the outcome variable attributable to person-related and situational differences. The ICC for the five dimensions of functions of music listening were: 0.48 for intellectual stimulation, 0.42 for mind wandering & emotional involvement, 0.40 for motor synchronization & enhanced well-being, 0.42 for updating one's musical knowledge, and 0.51 for killing time & overcoming loneliness. Across the five dimensions, on average 44% of the variance of functions of music listening was attributable to between-person differences, whereas 56% were attributable to within-person differences between situations. The ICC for the eight musical characteristics were 0.10 for *calming–exciting*, 0.10 for *slow–fast*, 0.17 for *sad–happy*, 0.22 for *less melodic–very melodic*, 0.24 for *less rhythmic–very rhythmic*, 0.16 for *simple–complex*, 0.08 for *peaceful–aggressive*, and 0.22 for *less intense–very intense*. Across all characteristics, between-person differences on average accounted for 16% of variance, whereas within-person differences between situations accounted for 84% of variance. This means that music selection was influenced largely by situational factors. In contrast, reported functions of music showed higher between-person variance, but were still outweighed by within-subject variability.

### Most Important Predictors of Music Selection

Models resulting from the analysis of the initial three steps A to C are presented in the Supplementary Material, and the models of the final analysis step D—representing the most comprehensive models—are shown in Table 1. Modeling results revealed that time (time of day, weekday vs. weekend) strongly contributed to the prediction of functions of music listening (step B), but played a minor role in the prediction of music selection (steps A and D). A closer inspection of the comprehensive models including all potential predictors in Table 1 shows that situation-specific arousal, degree of attention, and functions of music listening proved to be most important in the prediction of music selected in a specific situation. The theory-based assumption (cf. Figure 1) that functions of music listening play a significant role in music selection was supported by the observation that almost all direct effects that were detected during step C were also selected when all potential covariates were included in the model during step D, while regression coefficients remained virtually identical. Furthermore, the percentile-Lasso almost exclusively selected situational (Level 1) predictors of music-selection behavior, whereas only very few person-related (Level 2) variables were selected, namely a few aggregated mood and function scores. None of the personality traits or musical sophistication scores contributed to the prediction of music selection. Although momentary activity contributed substantially to the prediction of functions of music listening during step B (activity was included in four out of five models) and was also selected in four out of eight models during step A, it was only of marginal importance during step D as it was only included in two out of eight models. This clearly indicates the potential mediating role of functions of music listening in the association of person- and situation-related variables with music selection.

**Table 1:** Fixed effects estimates (top) and standard deviations of random parameters (bottom) for models of the predictors of music selection.

**Parameter**	**Calming—**	**Slow—fast**	**Sad—happy**	**Less melodic—**	**Less rhythmic—**	**Simple—**	**Peaceful—**	**Less intense—**
	**exciting**			**very melodic**	**very rhythmic**	**complex**	**aggressive**	**very intense**
**FIXED EFFECTS**								
Intercept	4.15 (0.06)^***^	4.11 (0.06)^***^	2.45 (0.05)^***^	4.93 (0.06)^***^	4.58 (0.07)^***^	4.00 (0.06)^***^	3.26 (0.05)^***^	3.53 (0.06)^***^
**Level 1 (situational)**								
Time			0.03 (0.02)					
Time^2^			>−0.01 (< 0.01)					
Mood								
Valence			0.14 (0.02)^***^					
Arousal	0.11 (0.01)^***^	0.05 (0.01)^***^					0.06 (0.01)^***^	0.04 (0.02)^*^
Importance of mood			0.02 (0.02)					0.02 (0.02)
Attention	0.14 (0.02)^***^	0.07 (0.02)^***^	−0.04 (0.02)^*^				0.08 (0.02)^***^	0.14 (0.02)^***^
Activity^a^								
Pure music listening			−0.18 (0.06)^**^					0.07 (0.06)
Housework			−0.12 (0.07)					0.13 (0.07)^*^
Working & studying			−0.17 (0.07)^*^					0.03 (0.07)
Coping with emotions			−0.53 (0.16)^***^					−0.03 (0.15)
Exercise			−0.68(0.14)^***^					0.16 (0.14)
Social activity			−0.11 (0.10)					0.12 (0.10)
Party			−0.70 (0.26)^*^					−0.29 (0.26)
Making Music			0.15 (0.17)					−0.35 (0.17)^*^
Relaxing & falling asleep			−0.06 (0.11)					−0.19 (0.11)
Personal hygiene			−0.15 (0.12)					0.03 (0.11)
Other activities			−0.10 (0.12)					0.17 (0.12)
Presence of others^b^								
Others present & no interaction			−0.06 (0.06)				0.05 (0.05)	
Others present & interaction			0.03 (0.07)				−0.25 (0.06)^***^	
Functions of music listenning								
Intellectual stimulation	−0.23 (0.02)^***^	−0.23 (0.02)^***^	−0.19 (0.02)^***^	0.20 (0.02)^***^		0.36 (0.02)^***^	−0.15 (0.02)^***^	0.24 (0.02)^***^
Mind wandering & emotional involvement	−0.08 (0.02)^***^	−0.10 (0.02)^***^		0.12 (0.02)^***^			−0.14 (0.02)^***^	0.12 (0.02)^***^
Motor synchronization & enhanced well-being	0.52 (0.02)^***^	0.47 (0.02)^***^	0.44 (0.02)^***^	−0.06 (0.02)^**^	0.33 (0.02)^***^		0.32 (0.02)^***^	0.04 (0.02)^*^
Updating ones musical knowledge				−0.09 (0.02)^***^				−0.08 (0.02)^***^
Killing time & overcoming loneliness			−0.05 (0.02)^*^				−0.12 (0.02)^***^	−0.06 (0.02)^*^
**Level 2 (person-related)**								
Mood								
Valence			0.22 (0.06)^***^					
Presence of others^b^								
Others present & no interaction								0.62 (0.26)^*^
Others present & interaction								0.09 (0.34)
Intensity of music preference								
Musical taste								
Rock & Metal								0.02 (0.04)
Functions of music listenning								
Intellectual stimulation			−0.20 (0.06)^**^					0.21 (0.07)^**^
Mind wandering & emotional involvement			−0.08 (0.06)					0.20 (0.06)^**^
Motor synchronization & enhanced well-being			0.43 (0.06)^***^					
Updating ones musical knowledge								−0.23 (0.07)^**^
Killing time & overcoming loneliness								
**RANDOM PARAMETERS**								
**Level 2 (*****SD*****)**								
Intercept/intercept	0.64^***^	0.54^***^	0.46^***^	0.64^***^	0.76^***^	0.58^***^	0.50^***^	0.53^***^

*Analysis step D (see text for details)*.

*Standard error in parentheses. SD = standard deviation*.

*Based on a total of 2,674 daily life assessment from N = 119 participants. The table only includes predictors that at least have been selected in one of the eight models. For a full list of included variables in the selection process of the percentile-Lasso see equations 1 and 2*.

a *Activity comprised 12 categories, reference category: being on the move*.

b *Presence of others comprised 3 categories, reference category: alone*.

* p < 0.05,

** p < 0.01,

*** *p < 0.001*.

### Mediation Analysis

**Figure 3:**
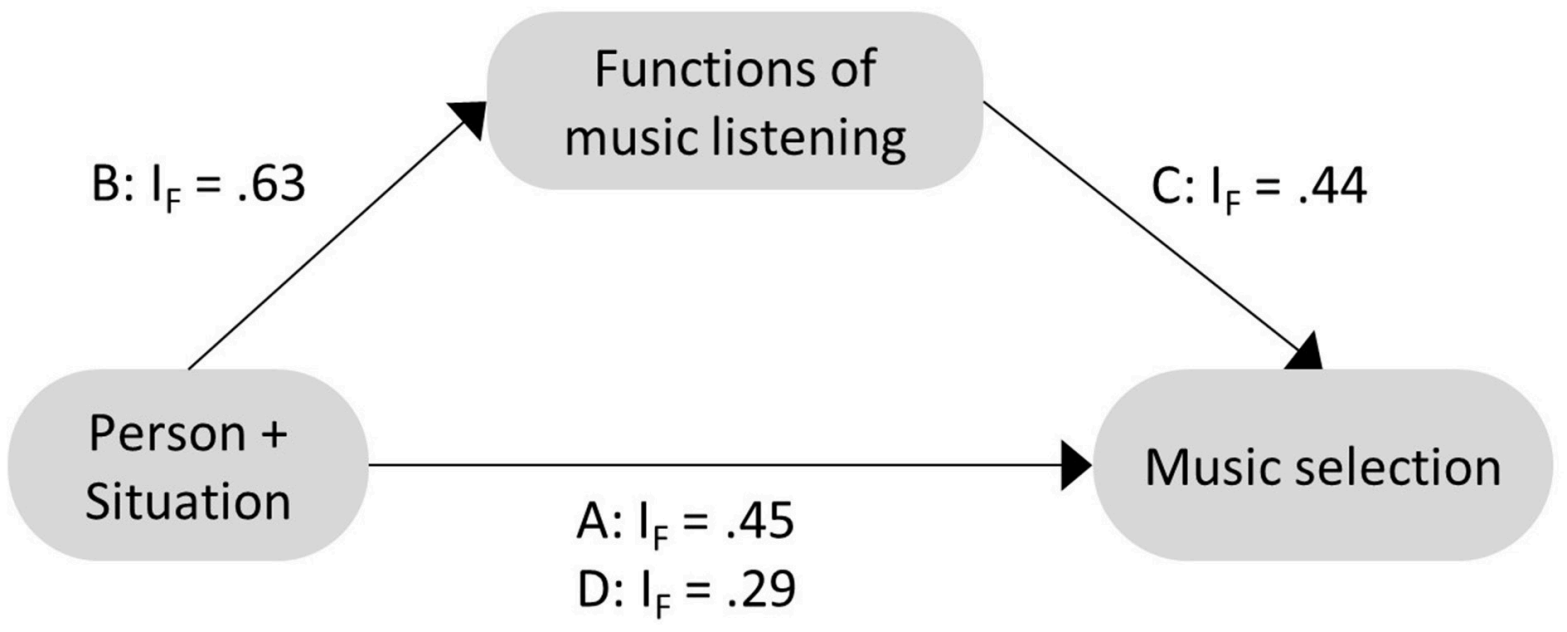
Summary of holistic mediation analysis using the percentile-Lasso. I_F_ is a consistency indicator summarizing all included person and situation predictors across all respective models for each step of analysis A–D (see text for details).

The mediation hypothesis was further supported by the consistency indicator *I*_*F*_ shown in Figure 3. The decrease of *I*_*F*_ from steps A to D clearly indicated that some of the variables selected in step A were no longer selected in step D in which functions of music listening were included as covariates. Exclusion of direct effects in the presence of potential mediators can be interpreted as full mediation.

**Figure 4:**
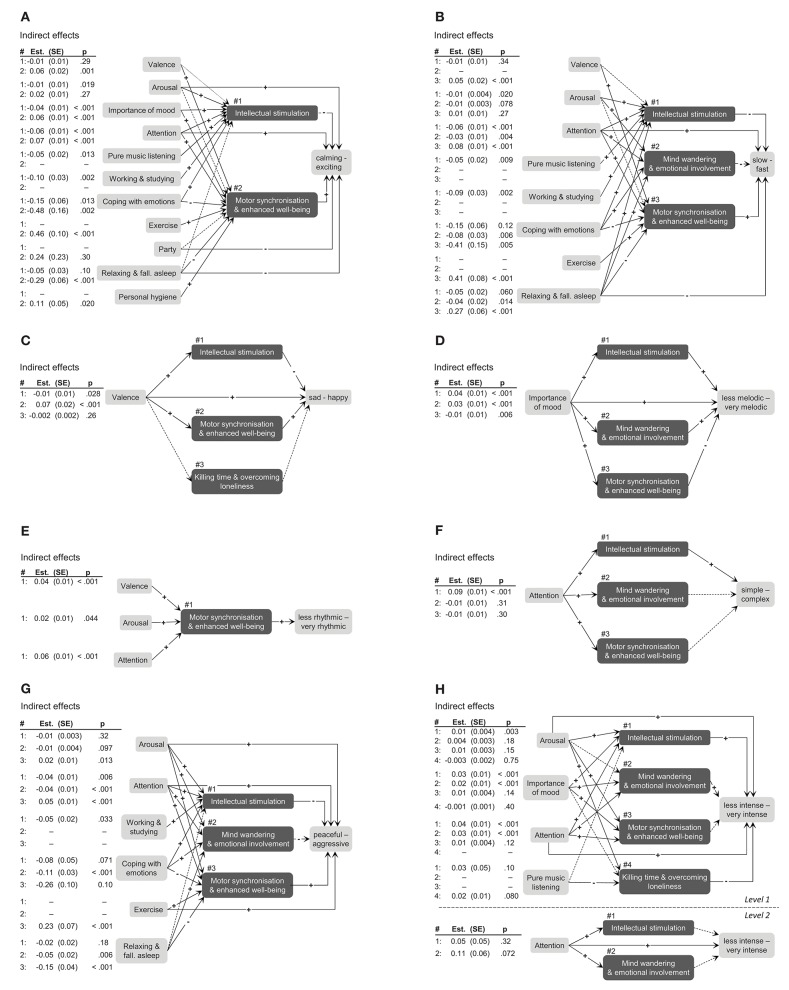
Multilevel mediation models for musical characteristics selected. **(A–G)** represent 1-1-1 mediations, **(H)** shows 1-1-1 and 2-2-2 mediations. Variables included in the analyses where selected by the percentile-Lasso (see text for details). Plus and minus signs indicate direction of effects. Solid lines represent significant effects and dashed lines represent non-significant effects (α = 0.05). Indirect effect parameters and tests from mediation models are shown in the tables left to each subfigure. Direct effects parameters are included in the detailed figures of the Supplementary Material.

Figure 4 depicts all mediation models including indirect effects (see Supplementary Material for detailed model summaries including coefficients of all effects). The results of these analyses revealed a similar pattern as seen above, but provided further insights, particularly with regard to direct effect tests and residual paths. For example, when people reported to be in a positive mood (valence), felt higher arousal, and payed higher attention to the music compared with their individual average, they tended to listen to music because they could move to it and feel fitter. This mediating functional state was in turn associated with a higher tendency to listen to rhythmic music (Figure 4, Model E). None of the three variables in this model showed a significant residual direct effect on the selection of rhythmic music, but all indirect effects were statistically significant. In another mediation model, the model for predicting selection of *sad–happy* music (Figure 4, Model C) revealed detailed findings on the broad effects of valence at the moment of the decision to listen to music. The model included an indirect positive effect of valence on happy music via motor synchronization and enhanced well-being, which was also found for choosing rhythmic (Model E), exciting (Model A), and fast music (Model B). In addition, there was a residual direct positive effect of valence on the selection of happy music, which reflects mood congruent selection of happy music. Moreover, the significant indirect path via intellectual stimulation demonstrates that people were more likely to listen to music for intellectual stimulation when they were in a positive mood, but tended to select *sad* music in that case. Such differentiated insights in specific associations including opposing directions within a mediation path were found for several models (Models A, B, C, D, and H). The mediation paths found in this study highlight both the general importance and the role of functions of music listening as a mediator in music-selection behavior. Overall, 52 (69%) of the 72 indirect effects tested throughout the eight models were significant (see Table 2).

**Table 2:** Number of significant indirect effects of MSEM mediation analyses.

**Outcome**	**Number of estimated indirect effects**	**Number of significant indirect effects**	**Percentage of significant indirect effects (%)**
Calming–exciting	17	13	76
Slow–fast	17	13	76
Sad–happy	3	2	67
Less melodic–very melodic	3	3	100
Less rhythmic–very rhythmic	3	3	100
Simple–complex	3	1	33
Peaceful–aggressive	14	11	79
Less intense–very intense	15	6	40
Sum	75	52	69

### Individual Differences of Situational Effects on Music Selection

Table 3 shows the re-estimation of the models derived from step D including random slopes for those predictors that yielded significant fixed effects in the percentile-Lasso model output. Many of the random parameters revealed significant individual variability around the overall mean effect. This variability was found consistently across all of the eight models of music-selection behavior. Overall, 24 (60%) of the 40 random parameters of this analysis step were statistically significant. Particularly, the three functions of music listening intellectual stimulation, mind wandering & emotional involvement, and motor synchronization & enhanced well-being consistently showed individual variability in their association with music-selection behavior. Estimations of fixed effects were only affected marginally by the inclusion of random effects, and almost all fixed effects remained significant. This indicates that general trends can be detected reliably, but some individuals deviate from this overall trend. Such individual deviations from overall trends offer the opportunity to investigate person-related moderators in future studies.

**Table 3:** Fixed effects estimates (top) and standard deviations of random parameters (bottom) for models of the predictors of music selection.

**Parameter**	**Calming—**	**Slow—fast**	**Sad—happy**	**Less melodic—**	**Less rhythmic—**	**Simple—**	**Peaceful—**	**Less intense—**
	**exciting**			**very melodic**	**very rhythmic**	**complex**	**aggressive**	**very intense**
**FIXED EFFECTS**								
Intercept	4.15 (0.06)^***^	4.12 (0.05)^***^	2.47 (0.36)^***^	4.93 (0.06)^***^	4.58 (0.07)^***^	4.00 (0.06)^***^	3.27 (0.05)^***^	3.53 (0.27)^***^
**Level 1 (situational)**								
Time			0.02 (0.02)					
Time^2^			>−0.01 (< 0.01)					
Mood								
Valence			0.12 (0.02)^***^					
Arousal	0.11 (0.02)^***^	0.04 (0.02)					0.06 (0.02)^**^	0.04 (0.02)^*^
Importance of mood			0.01 (0.02)					0.01 (0.02)
Attention	0.14 (0.03)^***^	0.07 (0.02)^**^	−0.03 (0.02)				0.07 (0.03)^**^	0.14 (0.02)^***^
Activity^a^								
Pure music listening			−0.21 (0.10)^*^					0.05 (0.07)
Housework			−0.19 (0.10)^*^					0.09 (0.09)
Working & studying			−0.23 (0.10)^*^					0.01 (0.08)
Coping with emotions			−0.59 (0.22)^*^					0.01 (0.17)
Exercise			−0.71 (0.17)^***^					0.13 (0.15)
Social activity			−0.15 (0.11)					0.13 (0.10)
Party			−0.60 (0.33)					−0.47 (0.29)
Making Music			−0.10 (0.21)					−0.50 (0.22)^*^
Relaxing & falling asleep			−0.10 (0.12)					−0.16 (0.10)
Personal hygiene			−0.16 (0.13)					−0.03 (0.11)
Other activities			−0.12 (0.11)					0.11 (0.09)
Presence of others^b^								
Others present & no interaction			−0.09 (0.07)				0.04 (0.07)	
Others present & interaction			>−0.01 (0.08)				−0.21 (0.08)^*^	
Functions of music listening								
Intellectual stimulation	−0.21 (0.04)^***^	−0.22 (0.04)^***^	−0.16 (0.04)^***^	0.21 (0.04)^***^		0.33 (0.04)^***^	−0.12 (0.04)^**^	0.24 (0.04)^***^
Mind wandering & emotional involvement	−0.07 (0.03)^*^	−0.09 (0.03)^**^		0.12 (0.02)^***^			−0.14 (0.03)^***^	0.13 (0.03)^***^
Motor synchronization & enhanced well-being	0.52 (0.04)^***^	0.48 (0.03)^***^	0.44 (0.03)^***^	−0.04 (0.03)	0.34 (0.03)^***^		0.32 (0.04)^***^	0.08 (0.03)^**^
Updating ones musical knowledge				−0.09 (0.03)^**^				−0.05 (0.03)
Killing time & overcoming loneliness			−0.06 (0.03)				−0.09 (0.03)^*^	−0.04 (0.03)
**Level 2 (person-related)**								
Mood								
Valence			0.22 (0.06)^***^					
Presence of others^b^								
Others present & no interaction								0.62 (0.26)^*^
Others present & interaction								0.09 (0.33)
Intensity of music preference								
Musical taste								
Rock & Metal								0.02 (0.04)
Functions of music listening								
Intellectual stimulation			−0.21 (0.06)^**^					0.21 (0.07)^**^
Mind wandering & emotional involvement			−0.07 (0.06)					0.20 (0.06)^**^
Motor synchronization & enhanced well-being			0.43 (0.06)^***^					
Updating ones musical knowledge								−0.23 (0.07)^**^
Killing time & overcoming loneliness								
**RANDOM PARAMETERS**								
**Level 2 (SD)**								
Intercept/intercept	0.57^***^	0.50^***^	0.44^***^	0.63^***^	0.73^***^	0.56^***^	0.45^***^	0.53^***^
Valence/valence			< 0.01					
Arousal/arousal	0.07	0.12^*^					< 0.01	0.05
Attention/attention	0.17^***^	0.11	0.10^*^				0.10	0.13^***^
Pure music listening/pure music listening			0.34					
Household/household								0.31^*^
Working & studying/working & studying			0.37^*^					
Coping with emotions/coping with emotions			0.53					
Exercise/exercise			< 0.01					
Party/party			< 0.01					
Making music/making music								0.43
Others present & interaction/others present & interaction							< 0.01	
Intellectual stimulation/intellectual stimulation	0.16	0.18^**^	0.24^***^	0.25^***^		0.28^***^	0.14	0.28^***^
Mind wandering & emotional involvement/mind wandering & emotional involvement	0.18^***^	0.17^***^		0.07			0.14^**^	0.12^*^
Motor synchronization & enhanced well-being/motor synchronization & enhanced well-being	0.22^***^	0.20^***^	0.21^***^	0.20^***^	0.21^***^		0.20^***^	0.13^**^
Updating ones musical knowledge/updating ones musical knowledge				0.13^*^				0.17^***^
Killing time & overcoming loneliness/killing time & overcoming loneliness			0.11				0.11	< 0.01

*Models Include Random Slopes*.

*Standard error in parentheses. SD = standard deviation*.

*Based on a total of 2,674 daily life assessment from N = 119 participants. The table only includes predictors that at least have been selected in one of the eight models. Predictors were selected by the percentile-Lasso (see data analysis fordetails)*.

a *Activity comprised 12 categories, reference category: being on the move*.

b *Presence of others comprised 3 categories, reference category: alone*.

* p < 0.05.

** p < 0.01.

*** *p < 0.001*.

### Comparison of Results With Greb et al. (2018b)

As we largely measured the same variables and used the same statistical-learning method for data analysis and variable selection (percentile-Lasso) as in an earlier study (Greb et al., 2018b), we had the opportunity to compare results between two studies that differ in their participant samples and assessment methods (retrospective online survey vs. momentary assessments in daily life). Table 4 shows comparisons of variance components and fixed effects consistently selected in both studies. ICC values were virtually identical for four of the outcome variables but deviated for *simple–complex* and *peaceful–aggressive*. Exclusively situational (i.e., within-subject centered) predictors were congruently selected across both studies. For the functions of music listening, intellectual stimulation and motor synchronization & enhanced well-being, results were most consistent. For example, when people reported listening to music for intellectual stimulation, they tended to select slower, more melodic, less happy, more peaceful, and more complex music in both studies. However, even though all effects shared the same directions, effect sizes of the current study were smaller when compared with the effects of the retrospective online study. As all effects shown in Table 4 consistently contributed to the prediction of music selection of unseen persons, these effects can be regarded as highly robust and reliable.

**Table 4:** Comparison of key indicators (top) and predictor variables (bottom) of retrospective online study by Greb et al. (2018b) and the current ESM study.

	**Less melodic—**		**Less rhythmic—**		**Slow—fast**		**Sad—happy**		**Simple—**		**Peaceful—**	
	**very melodic**		**very rhythmic**						**complex**		**aggressive**	
	**RS**	**ESM**	**RS**	**ESM**	**RS**	**ESM**	**RS**	**ESM**	**RS**	**ESM**	**RS**	**ESM**
ICC	0.28	0.22	0.22	0.24	0.09	0.10	0.18	0.17	0.29	0.16	0.32	0.09
# Predictors	1	4	1	1	11	5	7	14	13	1	10	7
**PARAMETERS**	**PREDICTORS CONSISTENTLY SELECTED IN BOTH STUDIES: ESTIMATES**											
**Situational predictors**												
Intellectual Stimulation	0.59 (0.06)	0.20 (0.02)			−0.49 (0.07)	−0.23 (0.02)	−0.09 (0.08)	−0.19 (0.02)	0.61 (0.08)	0.36 (0.02)	−0.25 (0.07)	−0.15 (0.02)
Motor sync. & enhanced well-being			0.79 (0.05)	0.33 (0.02)	0.91 (0.06)	0.47 (0.02)	0.73 (0.07)	0.44 (0.02)			0.59 (0.07)	0.32 (0.02)
Killing time & overcoming loneliness											0.14 (0.07)	−0.12 (0.02)
Attention					0.06 (0.03)	0.07 (0.02)	0.03 (0.03)	−0.04 (0.02)			0.06 (0.03)	0.08 (0.02)
Valence^a^							0.21 (0.03)	0.14 (0.02)				
Arousal^a^					0.10 (0.03)	0.05 (0.01)					0.07 (0.03)	0.06 (0.01)

*Standard errors in parentheses. RS = Retrospective online study by Greb et al. (2018b). ESM = Current study using the experience sampling method. The table only includes predictors which have been selected in both studies and were measured identically*.

a *Effects of valence and arousal are reported separately in Greb et al. (2018b)*.

## Discussion

The current study investigated music selection in daily life by using daily life research and statistical learning methods. Our first aim was to investigate to what extent person-related and situational variables influence music selection. Findings demonstrated that characteristics of music chosen in daily life were influenced largely by the situation a person resides in, with 84% of variance being attributable to situational factors. The predominance of situational influence is consistent with Greb et al. (2018b) whose results revealed virtually identical ICC values for four outcome variables. For the two variables *simple–complex* and *peaceful–aggressive*, the ICC values were considerably smaller in the present study. This difference might be explained by the fact that the results of Greb et al. (2018b) were based on retrospective self-reports of three listening situations collected in an online study. The two variables for which the differences occurred are probably most susceptible to response bias due to self-perception processes. For example, people perceiving themselves as highly intellectual music connoisseurs are more likely to report situations in which they listen to complex music when being asked retrospectively. As momentary assessments are less susceptible to such biasing factors (Schwarz, 2012), they very likely represent situational influences more accurately. In addition, the daily life research design employed here reflects a much more comprehensive representation of participants' daily lives as compared with reports of just three situations. Our results concerning the ICC values of *sad–happy* and *calming–exciting* are very similar to those of Randall and Rickard (2017a) who also used daily-life assessments and measured music selection via valence and arousal. In summary, the high situational variability of music-listening behavior revealed in the current study should initiate a shift from research on individual differences to situational influences and potential interactions on music-selection behavior. However, investigating the significance of situational variability means that studies need to be designed in a way that ensures ecological validity of observations, limits recall bias due to retrospective assessment, provides sufficiently large and heterogeneous samples of situations, and measures a wide variety of potentially relevant variables. In addition, suitable statistical methods need to be applied that can be used to separate within-subject from between-subject associations, and properly deal with a large number of predictors in the model. We believe that the sampling design and analysis procedure used in this study (cf. Figure 2) could serve as a template for a shift of research focus toward situational factors in music-listening behavior.

Our second aim was to identify the key variables involved in music selection. Our multi-step analytic plan revealed a detailed pattern of findings for a broad set of relevant variables. The finding that activity was important in predicting functions of music listening is consistent with previous research also revealing activity as very important in predicting listening functions or music perception in daily life (Krause and North, 2017b; Randall and Rickard, 2017a; Greb et al., 2018a). Regarding the aggregated situational variables, results showed that presence of others was the only situational variable that yielded an effect on level 2. This effect indicates that individuals with a propensity to listen to music in the presence of others while not communicating with them, tend to select more intense music. This is largely in line with (Tarrant et al., 2000), who also found individual differences in why people listen to music with regard to the presence of others (i.e., people with a propensity to listen to music alone tended to use music for emotional needs). The insight that, when controlling for the largest possible set of covariates, situation-specific attention, arousal, and functions of music listening were most important in predicting music selection is largely in line with findings by Greb et al. (2018b). Many of the direct effects that were revealed in the first steps of our analysis dropped out when controlling for functions of music listening. This supports our theoretical model proposing a mediating role of functions of music listening for the association between person- and situation-related predictor variables and music-selection behavior.

Clarifying the mediating role of functions of music listening in music selection was our third aim. Several analyses supported the mediation hypothesis. First, our consistency indicator clearly suggested several full mediations. Second, our findings demonstrated that in many cases (69% of tested indirect effects) functions of music listening acted as mediators on the selection of music with specific characteristics. It is important to mention that the mediation processes found in our study were exclusively located on the situational level (Level 1). Momentary mood, attention, and activity largely determined why participants listened to music, and the specific functions ultimately predicted which music participants selected. We did not find any significant mediation effects on the between-subject level (Level 2). The large within-subject variance of musical characteristics selected might explain this absence of between-subject mediations. The novel findings on within-subject mediations help to understand the important role of functions of music listening in music selection that would have been neglected by an analysis strategy strictly focusing on direct effects. For example, our results related to the direct effect of valence on the selection of *happy–sad* music confirm mood-congruent selection of music (Thoma et al., 2012; Randall and Rickard, 2017a), whereas the indirect effect via intellectual stimulation demonstrates mood-incongruent selection of music. These findings for the first time clearly differentiate the complex processes involved when people select music in daily life.

Our fourth aim was to investigate individual variations of situational effects identified in the previous analyses. Here, findings demonstrated that many associations significantly varied around the estimated mean effect. The three functions of intellectual stimulation, mind wandering & emotional involvement, and motor synchronization & enhanced well-being most consistently showed individual deviations. For example, this indicates that people generally tend to select faster, happier, less melodic, more rhythmic, more aggressive, more intense, and more exciting music to move to and enhance their well-being, but individuals do deviate from these overall trends. Hence, future research should investigate which person-related factors might explain the individual variability found here. One analysis strategy might be to add cross-level interaction parameters. Such an analysis could focus on two associations outlined by our theoretical model (Figure 1): individual variability in the association between situational variables and functions of music listening and music-selection behavior. In addition, moderated mediation models could be used to check whether person-related variables are capable of explaining individual variation in the mediation of the association between predictors and music-selection behavior by functions of music listening. This approach would also provide an opportunity to integrate person-related variables more precisely into theoretical models of music-selection behavior. It might well be the case that very few direct effects of person-related variables on music selection exist, and that person-related variables primarily act as moderators. Once more, this would suggest a shift from exclusively investigating individual differences to interaction effects between situational and person-related variables on music-listening behavior.

Finally, we aimed to compare the results of this daily-life study to those of a recent study on music selection that was very similar in terms of theoretical background and statistical analysis but analyzed data from a retrospective online survey (Greb et al., 2018b). Besides the virtual identical ICC values discussed above, we found numerous effects going in the same direction. Exclusively situational predictors were selected congruently across both studies. Consensus was greatest for intellectual stimulation and motor synchronization & enhanced well-being. For example, findings consistently indicated that people tend to select more melodic, less fast, less happy, less aggressive, and more complex music when they listen to music for intellectual stimulation. Although some effect sizes were largely identical, others differed in size with effects of this study being smaller than those obtained through the online study. This difference might be due to memory biases and a tendency to respond stereotypically in retrospective reports (Holmberg and Homes, 2012). The effects found across both studies can be regarded as highly robust and reliable. As in both studies, models were optimized to make predictions on unseen participants, these effects can be used to guide stimulus selection for experimental research investigating specific functions or effects of music listening. In addition, the similarity of results highlights the power of using statistical learning methods, the percentile-Lasso in this case, for reliable variable selection. Despite broad congruency between the two studies, some differences were evident that mainly concern the selection of person-related predictors. In the online study, musical taste factors were found to be important predictors—being selected in three out of six models—but in the current study, only one musical taste factor was selected in one out of eight models. As already discussed above, *simple–complex* and *peaceful–aggressive* showed different ICC values across both studies with values of the online study being considerably larger. This further supports the point made above that participants in the online study might have reported stereotypical situations that match their attitudes and beliefs, such as musical taste, whereas in the daily life study, the behavioral report was much less biased. Hence, the current findings clearly reject the notion that musical taste is significantly associated with the selection of certain musical characteristics.

None of the Big Five personality traits was selected as a predictor of music-selection behavior in the current study. This is consistent with Schäfer and Mehlhorn (2017) who showed that personality traits cannot substantially account for differences between individuals in musical taste and preferences. However, Big Five personality traits might be too broad to predict fine-grained behavior such as music selection. Future research might investigate if facets of Big Five personality traits, which represent specific and unique aspects underlying the broad personality traits, are better predictors of music selection.

Our study includes several notable limitations. First, music selection was measured based on subjectively perceived musical characteristics based on a particular conceptualization of music-selection behavior. Convergence with objective measures, such as musical features, obtained by music information retrieval methods would provide interesting comparative data for some of the current findings. In particular, this comparison might reveal whether the perception of musical characteristics is congruent with objective characteristics or if subjective perception is influenced by the situation as well. Music-selection behavior could also be conceptualized via musical styles selected, which might yield a different pattern of results than that found here. A style-based conceptualization would help to clarify if people predominantly select and listen to their favorite styles of music but at the same time adjust their specific musical choices (i.e., characteristics of the music) within their favorite styles to the situation they reside in. Hence, future research should try to model these different conceptualizations simultaneously to best reflect interdependencies and isolate effects of individual variables in the context of the complex entirety of potentially relevant variables.

Second, not accounting for covariations of outcome variables constitutes another limitation. This restriction is due to a lack of software solutions that could be used to perform a Lasso regression in a multivariate multilevel model framework. It should be noted that a single multivariate model might yield slightly different results.

Third, participants had the possibility to skip the questionnaire by reporting that they did not listen to music in a specific situation. This might be related to their current mood or some other unknown variable but cannot be investigated further with the given data.

Lastly, our findings are based on a specific sample of young people who frequently listen to music and are familiar with digital technologies. These “digital natives” grew up with technologies that enable situation-specific music selection. Future studies should also including infrequent music listeners with greater age variability. Nevertheless, the fact that we found a large overlap of effects between the present and an earlier study that did not focus on frequent music listeners suggests that our results are reliable.

The current study investigated music-selection behavior in daily life from a comprehensive perspective, using representative and unbiased momentary samples from participants' everyday life and innovative statistical learning procedures suitable for this endeavor. We demonstrated that situational factors mainly drive music selection and identified detailed patterns of variables contributing to music-selection behavior. We also showed for the first time that functions of music listening act as mediators between the situation and music-selection behavior. Our study therefore contributes to the understanding of music-selection behavior, in particular how situational characteristics influence people's motives to listen to a particular kind of music and actual musical choices. These findings emphasize the importance of accounting for situational influences in music psychological research, and might contribute to improved music recommendation systems.

## Author Contributions

FG, JS, and WS designed the study. FG collected data. FG and WS developed and performed the statistical analysis. FG wrote the first draft of the manuscript. All authors interpreted data, reviewed, and edited the manuscript, and approved the final version of the manuscript.

### Conflict of Interest Statement

The authors declare that the research was conducted in the absence of any commercial or financial relationships that could be construed as a potential conflict of interest.
