# Supplementary material for: Modeling Music-Selection Behavior in Everyday Life: A Multilevel Statistical Learning Approach and Mediation Analysis of Experience Sampling Data
Source: Front Psychol. 2019 Mar 19;10:390. doi: 10.3389/fpsyg.2019.00390 (PMC6433931; doi:10.3389/fpsyg.2019.00390)
Supplement: Supplementary file 1 [file Data_Sheet_1.PDF]

frontiers

Supplementary Material

Modeling Music-Selection Behavior in Everyday Life: A Multilevel Statistical Learning Approach and Mediation Analysis of Experience Sampling Data

Fabian Greb*, Jochen Steffens, Wolff Schlotz

*** Correspondence:** Fabian Greb: fabian.greb@aesthetics.mpg.de

Content

Page

1 Questionnaire answered on Smartphones during the 10 day course of the study 1
1.1 Section 1 [Situation] 1
1.2 Section 2 [Music] 2
1.3 Section 3 [Functions of music listening] 3
2 Model equations entered in the percentile-Lasso procedure 5
2.1 Step A 5
2.2 Step B 5
2.3 Step C 6
3 Modelling results 7
3.1 Step A 7
3.2 Step B 9
3.3 Step C 11
4 Detailed results of MSEM mediation analyses 12
5 References 15

1 Questionnaire answered on Smartphones during the 10 day course of the study

The questionnaire was presented and answered through movisensXS, Version 1.0.1 (movisens GmbH, 2015)

1. Do you currently listen to music?

□ Yes, I currently listen to music.

□ No, I currently do not listen to music.

1.1 Section 1 [Situation]

2. For how long you have been listening to music already? Please indicate the duration in minutes:

Free response

3. Please choose your current main activity [Activity][1]:

□ Pure music listening
□ Housework
□ Working / studying
□ Coping with emotions
□ Exercise
□ Social activity (e.g., eating or playing with friends)
□ Party
□ Making music
□ Relaxing / falling asleep
□ Being on the move (bus/ train/ car)
□ Personal hygiene
□ Other (none of the activity listed is appropriate)

4. Are there currently any other persons present? [Presence of others]

□ No, I am alone.

□ Yes, I am surrounded by others but do not interact or communicate with them.
□ Yes, I interact or communicate with other people.

[1] The categories were developed by Greb, Schlotz, and Steffens (2017). We included personal hygiene as an additional category based on feedback of pretesting the current study.

5. How did you choose the music? [Choice]

□ I freely choose a specific piece or album.
□ I did not choose the music.
□ I am listening to radio.
□ I am listening to music in a club.
□ I am listening to music at a concert.
□ I am listening to a playlist

6. How much control do you have in what you hear? [Choice interval]

No control 1 – 2 – 3 – 4 – 5 – 6 – 7 Total control

7. How was your mood at the moment you decided to listen to music? [Valence]

Bad 1 – 2 – 3 – 4 – 5 – 6 – 7 Good

8. How awake did you feel at the moment you decided to listen to music? [Arousal]

Tired 1 – 2 – 3 – 4 – 5 – 6 – 7 Awake

9. How important was your mood for your decision to listen to music? [Importance of mood]

Not at all 1 – 2 – 3 – 4 – 5 – 6 – 7 Very important

10. How much attention are you paying to the music? [Attention]

Little 1 – 2 – 3 – 4 – 5 – 6 – 7 A lot

1.2 Section 2 [Music]

11. How loud is the music?

Quiet 1 – 2 – 3 – 4 – 5 – 6 – 7 Loud

12. How much do you like the music?

I like it less 1 – 2 – 3 – 4 – 5 – 6 – 7 I like it a lot

13. Please name the composer/ artist if known:

Free response

14. Please name the title of the piece if known:

Free response

15. Please name the musical style if known:

Free response

16. Which characteristics does the music have? [Musical characteristics]

Calming	1 – 2 – 3 – 4 – 5 – 6 – 7	Exciting
Slow	1 – 2 – 3 – 4 – 5 – 6 – 7	Fast
Sad	1 – 2 – 3 – 4 – 5 – 6 – 7	Happy
Unfamiliar	1 – 2 – 3 – 4 – 5 – 6 – 7	Familiar
Less melodic	1 – 2 – 3 – 4 – 5 – 6 – 7	Very melodic
Less rhythmic	1 – 2 – 3 – 4 – 5 – 6 – 7	Very rhythmic
Simple	1 – 2 – 3 – 4 – 5 – 6 – 7	Complex
Peaceful	1 – 2 – 3 – 4 – 5 – 6 – 7	Aggressive
Less intense	1 – 2 – 3 – 4 – 5 – 6 – 7	Very intense
Instrumental	0 – 1	Vocal

1.3 Section 3 [Functions of music listening]

17. Why do you currently listen to music? [Functions of music listening]

… because it gives me intellectual stimulation. (I)
Not at all 1 – 2 – 3 – 4 – 5 – 6 – 7 Fully agree
… because it mirrors my feelings and moods. (II)
Not at all 1 – 2 – 3 – 4 – 5 – 6 – 7 Fully agree
… because it makes me feel fitter. (III)
Not at all 1 – 2 – 3 – 4 – 5 – 6 – 7 Fully agree
… because it addresses my sense of aesthetics. (I)
Not at all 1 – 2 – 3 – 4 – 5 – 6 – 7 Fully agree
…. because it puts fantastic images or stories in my head. (II)
Not at all 1 – 2 – 3 – 4 – 5 – 6 – 7 Fully agree
… because I can learn about new pieces. (IV)
Not at all 1 – 2 – 3 – 4 – 5 – 6 – 7 Fully agree
… because it enables me to kill time. (V)
Not at all 1 – 2 – 3 – 4 – 5 – 6 – 7 Fully agree
… because it helps me learn about myself. (I)
Not at all 1 – 2 – 3 – 4 – 5 – 6 – 7 Fully agree
… because it reminds me of certain periods of my life or past experiences. (II)
Not at all 1 – 2 – 3 – 4 – 5 – 6 – 7 Fully agree
… because it makes me feel connected to all people who like the same kind of music. (IV)
Not at all 1 – 2 – 3 – 4 – 5 – 6 – 7 Fully agree
… because I can move to the music. (III)
Not at all 1 – 2 – 3 – 4 – 5 – 6 – 7 Fully agree
… because I need it in the background while I do other things. (V)
Not at all 1 – 2 – 3 – 4 – 5 – 6 – 7 Fully agree

… because I want to inform myself about hits and trends. (IV)
Not at all 1 – 2 – 3 – 4 – 5 – 6 – 7 Fully agree
… because it enhances my mood
Not at all 1 – 2 – 3 – 4 – 5 – 6 – 7 Fully agree
… because it makes me feel less lonely. (V)
Not at all 1 – 2 – 3 – 4 – 5 – 6 – 7 Fully agree
… because I do it out of habit. *
Not at all 1 – 2 – 3 – 4 – 5 – 6 – 7 Fully agree

[Roman numerals in parentheses indicate which items belong to which factor.
Intellectual Stimulation (I), Mind Wandering & Emotional Involvement (II), Motor Synchronization & Enhanced Well-being (III), Updating One's Musical Knowledge (IV), Killing Time & Overcoming Loneliness (V)
These indicators were not part of the study and not shown to participants. For a detailed report on the construction of the inventory see Greb, Schlotz, and Steffens (2017).
*This item was not part of the inventory and was not analyzed in the current study]

The questionnaire was originally presented in German language and is available upon request.

2 Model equations entered in the percentile-Lasso procedure

2.1 Step A

Level 1 equation:

$$
\begin{aligned}
Y_{ij} = \ & \beta_{0j} + \beta_1 \text{ time}_{ij} + \beta_2 \text{ time}^2_{ij} + \beta_3 \text{ weekend}_{ij} + \beta_4 \text{ valenceC}_{ij} + \beta_5 \text{ arousalC}_{ij} \\
& + \beta_6 \text{ imp.of.moodC}_{ij} + \beta_7 \text{ attentionC}_{ij} + \beta_8 \text{ activity1C}_{ij} + \cdots + \beta_{18} \text{ activity11C}_{ij} \\
& + \beta_{19} \text{ presence.of.others1C}_{ij} + \beta_{20} \text{ presence.of.others2C}_{ij} + R_{ij}
\end{aligned}
$$

Level 2 equation:

$$
\begin{aligned}
\beta_{0j} = \ & \gamma_{00} + \gamma_{01} \text{ valenceM}_j + \gamma_{02} \text{ arousalM}_j + \gamma_{03} \text{ imp.of.moodM}_j + \gamma_{04} \text{ attentionM}_j \\
& + \gamma_{05} \text{ activity1M}_j + \cdots + \gamma_{015} \text{ activity11M}_j + \gamma_{016} \text{ presence.of.others1M}_j \\
& + \gamma_{017} \text{ presence.of.others2M}_j + \gamma_{018} \text{ sex}_j + \gamma_{019} \text{ age}_j \\
& + \gamma_{020} \text{ intensity.musicpreference}_j + \gamma_{021} \text{ musical.taste1}_j + \cdots \\
& + \gamma_{026} \text{ musical.taste6}_j + \gamma_{027} \text{ big5.1}_j + \cdots + \gamma_{031} \text{ big5.5}_j + \gamma_{032} \text{ gold.msi1}_j + \cdots \\
& + \gamma_{036} \text{ gold.msi5}_j + U_{0j}
\end{aligned}
$$

2.2 Step B

Level 1 equation:

$$
\begin{aligned}
Y_{ij} = \ & \beta_{0j} + \beta_1 \text{ time}_{ij} + \beta_2 \text{ time}^2_{ij} + \beta_3 \text{ weekend}_{ij} + \beta_4 \text{ valenceC}_{ij} + \beta_5 \text{ arousalC}_{ij} \\
& + \beta_6 \text{ imp.of.moodC}_{ij} + \beta_7 \text{ attentionC}_{ij} + \beta_8 \text{ activity1C}_{ij} + \cdots + \beta_{18} \text{ activity11C}_{ij} \\
& + \beta_{19} \text{ presence.of.others1C}_{ij} + \beta_{20} \text{ presence.of.others2C}_{ij} + R_{ij}
\end{aligned}
$$

Level 2 equation:

$$
\begin{aligned}
\beta_{0j} = \ & \gamma_{00} + \gamma_{01} \text{ valenceM}_j + \gamma_{02} \text{ arousalM}_j + \gamma_{03} \text{ imp.of.moodM}_j + \gamma_{04} \text{ attentionM}_j \\
& + \gamma_{05} \text{ activity1M}_j + \cdots + \gamma_{015} \text{ activity11M}_j + \gamma_{016} \text{ presence.of.others1M}_j \\
& + \gamma_{017} \text{ presence.of.others2M}_j + \gamma_{018} \text{ sex}_j + \gamma_{019} \text{ age}_j \\
& + \gamma_{020} \text{ intensity.musicpreference}_j + \gamma_{021} \text{ musical.taste1}_j + \cdots \\
& + \gamma_{026} \text{ musical.taste6}_j + \gamma_{027} \text{ big5.1}_j + \cdots + \gamma_{031} \text{ big5.5}_j + \gamma_{032} \text{ gold.msi1}_j + \cdots \\
& + \gamma_{036} \text{ gold.msi5}_j + U_{0j}
\end{aligned}
$$

2.3 Step C

Level 1 equation:

$$Y_{ij} = \beta_{0j} + \beta_1 \text{ intel. stimulatonC}_{ij} + \beta_2 \text{ mind. wanderingC}_{ij} + \beta_3 \text{ motor. synchronizationC}_{ij} + \beta_4 \text{ updating. musical. knowledgeC}_{ij} + \beta_5 \text{ killing. timeC}_{ij} + R_{ij}$$

Level 2 equation:

$$\beta_{0j} = \gamma_{00} + \gamma_{01} \text{ intel. stimulationM}_j + \gamma_{02} \text{ mind. wanderingM}_j + \gamma_{03} \text{ motor. synchronizationM}_j + \gamma_{04} \text{ updating. musical. knowledgeM}_j + \gamma_{05} \text{ killing. timeM}_j + U_{0j}$$

For Step A and C Y_{ij} denotes the expected musical characteristic selected by person *j* at situation *i*. For Step B Y_{ij} denotes the expected function of music listening used by person *j* at situation *i.* In all steps β_{0j} represents a participant-specific intercept. This intercept is modeled following the level 2 equation including all person-related variables. Within-subject effects are represented by the beta coefficients (β_1–β_{25}) while γ_{01}–γ_{041} represent between-subject effects. Capital letter C denotes within-subject centered variables while M denotes aggregated variables at person level. The terms R_{ij} and U_j denote residuals at level 1 and level 2.

3 Modelling results

3.1 Step A

Fixed Effects Estimates (Top) and Standard Deviation of Random Parameters (Bottom) for Models of the Predictors of Music Selection.

Parameter	calming – exciting	slow – fast	sad – happy	less melodic – very melodic	less rhythmic – very rhythmic	simple – complex	peaceful – aggressive	less intense – very intense
					Fixed Effects			
Intercept	1.38 (0.06)***	4.12 (0.06)***	4.44 (0.06)***	4.93 (0.06)***	4.58 (0.07)***	4.00 (0.06)***	3.26 (0.05)***	2.72 (0.06)***
Level 1 (situational)								
Mood								
Valence	0.06 (0.02)**	0.04 (0.02)	0.20 (0.02)***		0.06 (0.02)***			
Arousal	0.10 (0.02)***	0.04 (0.02)**			0.05 (0.02)**		0.05 (0.02)***	0.05 (0.02)***
Importance of mood	0.04 (0.01)*			0.09 (0.01)***				0.08 (0.01)***
Attention	0.12 (0.02)***	0.05 (0.02)**			0.06 (0.01)***	0.10 (0.01)***	0.02 (0.02)	0.23 (0.02)***
Activity[a]								
Pure music listening	-0.15 (0.06)**	-0.09 (0.06)**					-0.09 (0.06)	0.17 (0.06)**
Housework	0.07 (0.06)	0.05 (0.06)					-0.07 (0.07)	0.11 (0.06)
Working & studying	-0.38 (0.07)***	-0.36 (0.07)***					-0.42 (0.07)***	0.12 (0.07)
Coping with emotions	-0.50 (0.15)**	-0.99 (0.15)***					-0.38 (0.15)*	0.18 (0.15)
Exercise	0.67 (0.14)***	0.38 (0.14)**					0.81 (0.14)***	0.15 (0.14)
Social activity	0.01 (0.10)	0.04 (0.10)					-0.10 (0.10)	0.13 (0.10)
Party	-0.64 (0.26)*	-0.30 (0.26)					0.06 (0.26)	-0.42 (0.26)
Making Music	-0.29 (0.16)	-0.03 (0.16)					0.05 (0.16)	-0.02 (0.16)
Relaxing & falling asleep	-0.96 (0.10)***	-0.77 (0.10)***					-0.62 (0.11)***	-0.09 (0.10)
Personal hygiene	0.27 (0.11)*	0.14 (0.11)					0.01 (0.11)	-0.04 (0.11)
Other activities	-0.02 (0.12)	-0.17 (0.12)					-0.09 (0.12)	0.21 (0.12)
Presence of others[b]								
Others present & no interaction							-0.04 (0.06)	
Others present & interaction							-0.28 (0.07)***	
Level 2 (person-related)								
Mood								
Arousal	0.21 (0.07)**							
Attention	0.26 (0.07)***							0.44 (0.07)***
Musical taste								
Pop	0.10 (0.04)**							
					Random parameters			
Level 2								
Intercept/intercept (*SD*)	0.55***	0.54***	0.63***	0.64***	0.76***	0.58***	0.50***	0.56***

Note. Standard errors are in parentheses. SD = standard deviation.
Based on a total of 2674 daily life assessment from N = 119 participants. The table only includes predictors that at least have been selected in one of the eight models. For a full list of included variables in the selection process of the percentile-Lasso see model equations of Step A on page 5.
[a] activity comprised 12 categories, reference category: being on the move. [b] presence of others comprised 3 categories, reference category: alone. * p < .05. ** p < .01. *** p < .001.

3.2 Step B

Fixed Effects Estimates (Top) and Standard Deviation of Random Parameters (Bottom) for Models of the Predictors of Functions of Music Listening.

Parameter	Intellectual stimulation	Mind wandering & emotional involvement[a]	Motor synchronization & enhanced well-being	Updating ones musical knowledge[b]	Killing time & overcoming loneliness
			Fixed Effects		
Intercept	-0.67 (0.08)***	-2.22 (0.09)***	4.21 (0.09)***	2.30 (0.08)***	6.18 (0.08)***
Level 1 (situational)					
Indiv.Time			-0.02 (0.01)***		0.00 (0.02)
Indiv.Time²		0.001 (0.0003)**			-0.00 (0.00)
Weekend	-0.05 (0.04)	-0.07 (0.04)			-0.04 (0.04)
Mood					
Valence	0.03 (0.02)		0.12 (0.02)***		0.03 (0.02)
Arousal	0.05 (0.02)**	0.06 (0.02)***	0.03 (0.02)		0.03 (0.02)*
Importance of mood	0.13 (0.01)***	0.18 (0.01)***	0.12 (0.01)***		0.02 (0.01)
Attention	0.19 (0.02)***	0.22 (0.02)***	0.14 (0.02)***		
Activity[c]					
Pure music listening	0.30 (0.06)***	0.11 (0.06)	0.09 (0.06)		-0.28 (0.06)***
Housework	0.06 (0.06)	-0.12 (0.06)	0.51 (0.06)***		0.32 (0.07)***
Working & studying	0.41 (0.07)***	-0.10 (0.07)	-0.05 (0.07)		0.01 (0.06)
Coping with emotions	0.67 (0.15)***	0.63 (0.15)***	-0.84 (0.15)***		-0.46 (0.15)**
Exercise	-0.16 (0.14)	-0.25 (0.14)	1.10 (0.14)***		-0.03 (0.14)
Social activity	0.06 (0.10)	0.00 (0.10)	0.11 (0.10)		-0.04 (0.10)
Party	-0.29 (0.26)	-0.65 (0.26)*	0.74 (0.26)**		-0.32 (0.26)
Making Music	1.21 (0.16)***	0.12 (0.16)	0.03 (0.16)		-0.94 (0.16)***
Relaxing & falling asleep	0.25 (0.10)*	0.30 (0.11)**	-0.44 (0.10)***		-0.35 (0.11)***
Personal hygiene	-0.06 (0.11)	-0.28 (0.11)*	0.31 (0.11)**		0.34 (0.11)**
Other activities	0.21 (0.12)	-0.07 (0.12)	0.09 (0.12)		0.14 (0.12)
Presence of others[d]					
Others present & no interaction				-.20 (0.05)***	0.12 (0.06)*
Others present & interaction				.26 (0.06)***	-0.24 (0.07)***
Level 2 (person-related)					
Importance of mood	0.21 (0.08)**	0.27 (0.08)**			
Attention	0.40 (0.11)***	0.53 (0.12)***			
Age					-0.08 (0.02)***
Personality traits					
Openness to experience		0.56 (0.23)*			
Gold MSI					
Active engagement	0.29 (0.10)**				
			Random parameters		

Level 2 (*SD*)					
Intercept/intercept	0.85***	0.93***	0.91***	0.84****	0.87***

Note. Standard errors in parentheses. SD = standard deviation.
Based on a total of 2674 daily life assessment from N = 119 participants. The table only includes predictors that at least have been selected in one of the eight models. For a full list of included variables in the selection process of the percentile-Lasso see model equations of Step B on page 5.
[a] For this model Indiv.time² was excluded from the random effects test as it caused estimation problems. [b] For this factor the 95th percentile of the percentile-lasso revealed to be too conservative and did not select any variables. We used the 93rd percentile instead which was the nearest percentile for which a variable was selected. [c] activity comprised 12 categories, reference category: being on the move. [d] presence of others comprised 3 categories, reference category: alone.
* p < .05. ** p < .01. *** p < .001.

3.3 Step C

Fixed Effects Estimates (Top) and Standard Deviation of Random Parameters (Bottom) for Models of the Predictors of Music Selection.

Parameter	calming – exciting	slow – fast	sad – happy	less melodic – very melodic	less rhythmic – very rhythmic	simple – complex	peaceful – aggressive	less intense – very intense
				Fixed Effects				
Intercept	4.15 (0.06)***	4.12 (0.05)***	3.63 (0.05)***	4.93 (0.06)***	4.57 (0.07)***	3.95 (0.05)***	3.26 (0.05)***	3.83 (0.06)***
Level 1 (situational)								
Intellectual stimulation	-0.22 (0.02)***	-0.21 (0.02)***	-0.19 (0.02)***	0.20 (0.02)***		0.38 (0.02)***	-0.12 (0.02)***	0.28 (0.02)***
Mind wandering & emotional involvement		-0.09 (0.02)***		0.12 (0.02)***		-0.02 (0.02)	-0.12 (0.02)***	0.15 (0.02)***
Motor synchronization & enhanced well-being	0.56 (0.02)***	0.50 (0.02)***	0.44 (0.02)***	-0.06 (0.02)**	0.33 (.02)***	-0.02 (0.02)	0.31 (0.02)***	0.10 (0.02)***
Updating ones musical knowledge				-0.09 (0.02)***		-0.03 (0.02)		-0.10 (0.02)***
Killing time & overcoming loneliness			-0.04 (0.02)			-0.03 (0.02)		-0.06 (0.02)**
Level 2 (person-related)								
Intellectual stimulation			-0.22 (0.07)***			0.23 (0.06)***		0.20 (0.07)**
Mind wandering & emotional involvement			-0.11 (0.06)					0.22 (0.06)***
Motor synchronization & enhanced well-being			0.48 (0.06)***					
Updating ones musical knowledge						-0.10 (0.07)		-0.24 (0.07)***
Killing time & overcoming loneliness						-0.13 (0.06)*		
				Random parameter				
Level 2								
Intercept/intercept (SD)	0.64***	0.54***	0.49***	0.64***	0.76***	0.53***	0.50***	0.54***

Note. Standard errors in parentheses. SD = standard deviation.
Based on a total of 2674 daily life assessment from N = 119 participants. The table only includes predictors that have been selected by the percentile-Lasso. For details se model equations of Step C on page 6.

* $p < .05$. ** $p < .01$. *** $p < .001$.

4 Detailed results of MSEM mediation analyses

a)

#	Est.	(SE)	p
1:	-0.01	(0.01)	.29
2:	0.06	(0.02)	.001
1:	-0.01	(0.01)	.019
2:	0.02	(0.01)	.27
1:	-0.04	(0.01)	< .001
2:	0.06	(0.01)	< .001
1:	-0.06	(0.01)	< .001
2:	0.07	(0.01)	< .001
1:	-0.05	(0.02)	.013
2:	–		–
1:	-0.10	(0.03)	.002
2:	–		–
1:	-0.15	(0.06)	.013
2:	-0.48	(0.16)	.002
1:	–		–
2:	0.46	(0.10)	< .001
1:	–		–
2:	0.24	(0.23)	.30
1:	-0.05	(0.03)	.10
2:	-0.29	(0.06)	< .001
1:	–		–
2:	0.11	(0.05)	.020

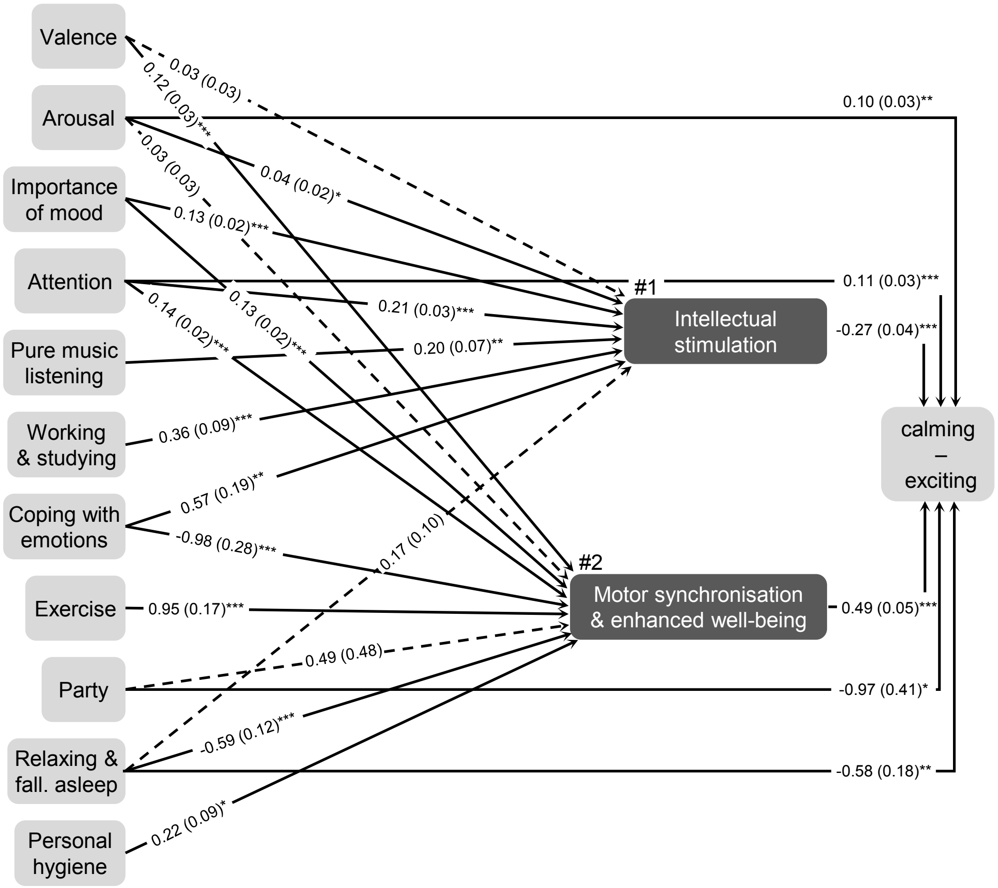

b)

#	Est.	(SE)	p
1:	-0.01	(0.01)	.34
2:	–		–
3:	0.05	(0.02)	< .001
1:	-0.01	(0.004)	.020
2:	-0.01	(0.003)	.078
3:	0.01	(0.01)	.27
1:	-0.06	(0.01)	< .001
2:	-0.03	(0.01)	.004
3:	0.08	(0.01)	< .001
1:	-0.05	(0.02)	.009
2:	–		–
3:	–		–
1:	-0.09	(0.03)	.002
2:	–		–
3:	–		–
1:	-0.15	(0.06)	0.12
2:	-0.08	(0.03)	.006
3:	-0.41	(0.15)	.005
1:	–		–
2:	–		–
3:	0.41	(0.08)	< .001
1:	-0.05	(0.02)	.060
2:	-0.04	(0.02)	.014
3:	.0.27	(0.06)	< .001

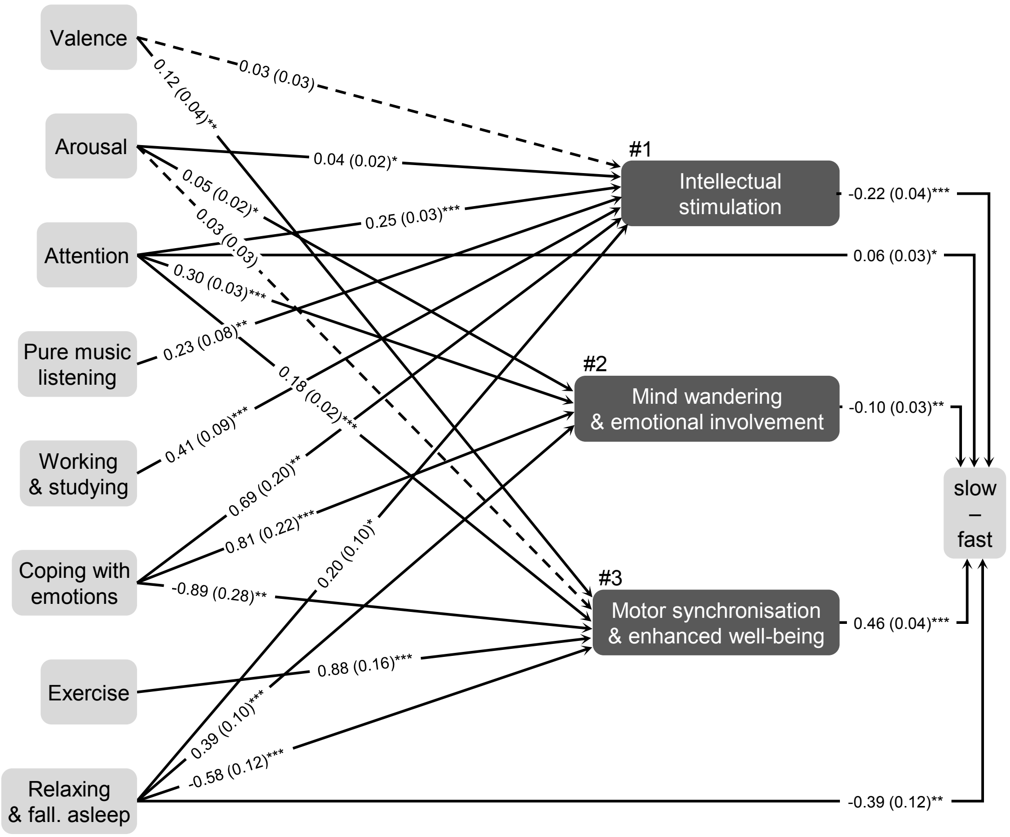

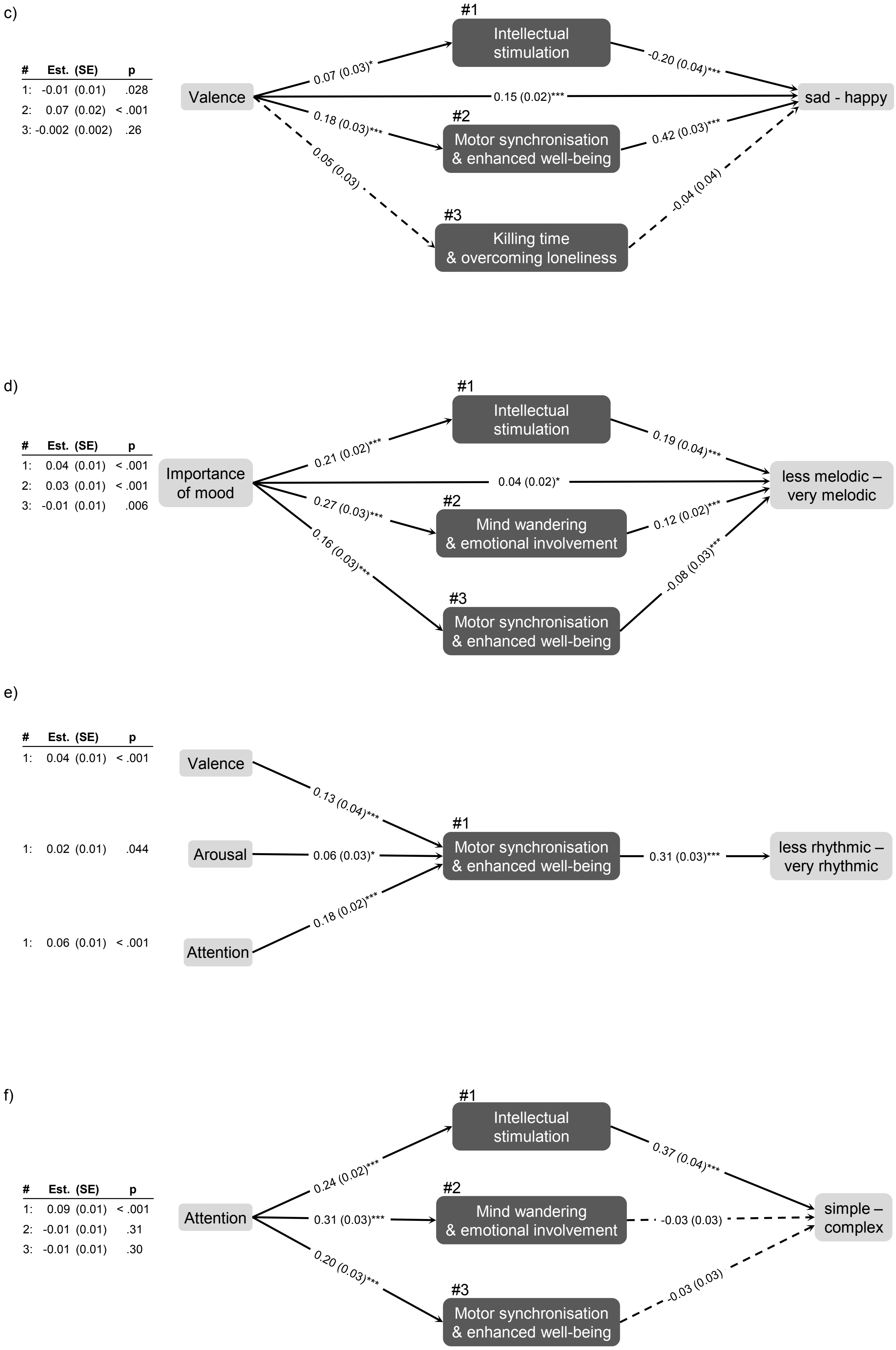
c)
Est. (SE) p
1: -0.01 (0.01) .028
2: 0.07 (0.02) < .001
3: -0.002 (0.002) .26
Valence
#1
Intellectual stimulation
#2
Motor synchronisation & enhanced well-being
#3
Killing time & overcoming loneliness
sad - happy
0.07 (0.03)*
0.15 (0.02)***
0.18 (0.03)***
0.05 (0.03)
-0.20 (0.04)***
0.42 (0.03)***
-0.04 (0.04)
d)
Est. (SE) p
1: 0.04 (0.01) < .001
2: 0.03 (0.01) < .001
3: -0.01 (0.01) .006
Importance of mood
#1
Intellectual stimulation
#2
Mind wandering & emotional involvement
#3
Motor synchronisation & enhanced well-being
less melodic – very melodic
0.21 (0.02)***
0.04 (0.02)*
0.27 (0.03)***
0.16 (0.03)***
0.19 (0.04)***
0.12 (0.02)***
-0.08 (0.03)***
e)
Est. (SE) p
1: 0.04 (0.01) < .001
1: 0.02 (0.01) .044
1: 0.06 (0.01) < .001
Valence
Arousal
Attention
#1
Motor synchronisation & enhanced well-being
less rhythmic – very rhythmic
0.13 (0.04)***
0.06 (0.03)*
0.18 (0.02)***
0.31 (0.03)***
f)
Est. (SE) p
1: 0.09 (0.01) < .001
2: -0.01 (0.01) .31
3: -0.01 (0.01) .30
Attention
#1
Intellectual stimulation
#2
Mind wandering & emotional involvement
#3
Motor synchronisation & enhanced well-being
simple – complex
0.24 (0.02)***
0.31 (0.03)***
0.20 (0.03)***
0.37 (0.04)***
-0.03 (0.03)
-0.03 (0.03)

g)

#	Est.	(SE)	p
1:	-0.01	(0.003)	.32
2:	-0.01	(0.004)	.097
3:	0.02	(0.01)	.013
1:	-0.04	(0.01)	.006
2:	-0.04	(0.01)	< .001
3:	0.05	(0.01)	< .001
1:	-0.05	(0.02)	.033
2:	–		–
3:	–		–
1:	-0.08	(0.05)	.071
2:	-0.11	(0.03)	< .001
3:	-0.26	(0.10)	0.10
1:	–		–
2:	–		–
3:	0.23	(0.07)	< .001
1:	-0.02	(0.02)	.18
2:	-0.05	(0.02)	.006
3:	-0.15	(0.04)	< .001

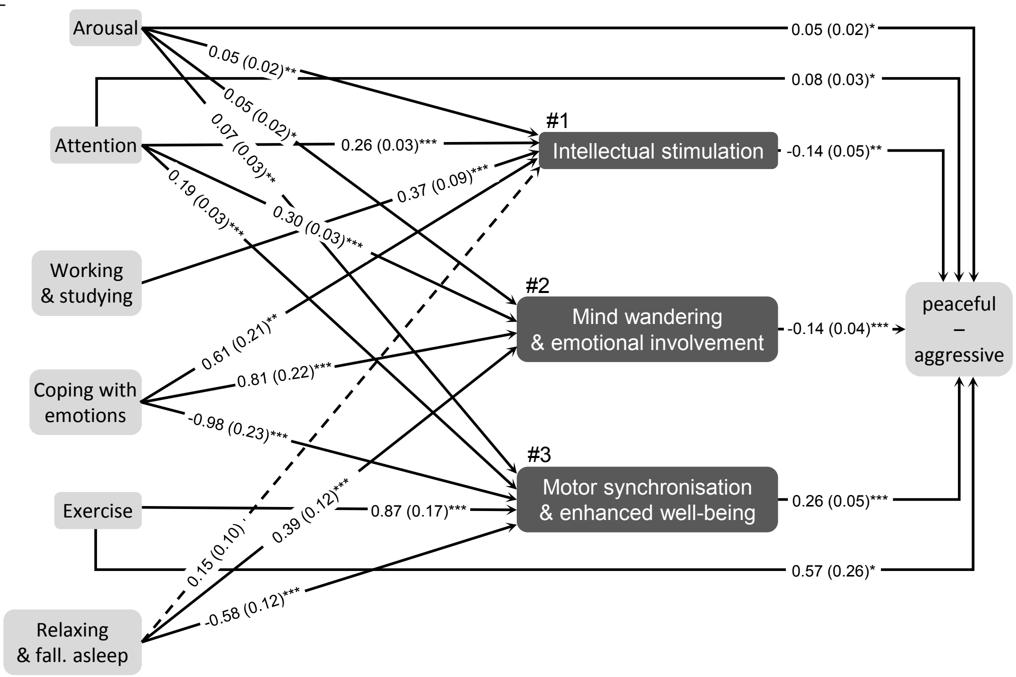

h)

#	Est.	(SE)	p
1:	0.01	(0.004)	.003
2:	0.004	(0.003)	.18
3:	0.01	(0.003)	.15
4:	-0.003	(0.002)	0.75
1:	0.03	(0.01)	< .001
2:	0.02	(0.01)	< .001
3:	0.01	(0.004)	.14
4:	-0.001	(0.001)	.40
1:	0.04	(0.01)	< .001
2:	0.03	(0.01)	< .001
3:	0.01	(0.004)	.12
4:	–		–
1:	0.03	(0.05)	.10
2:	–		–
3:	–		–
4:	0.02	(0.01)	.080

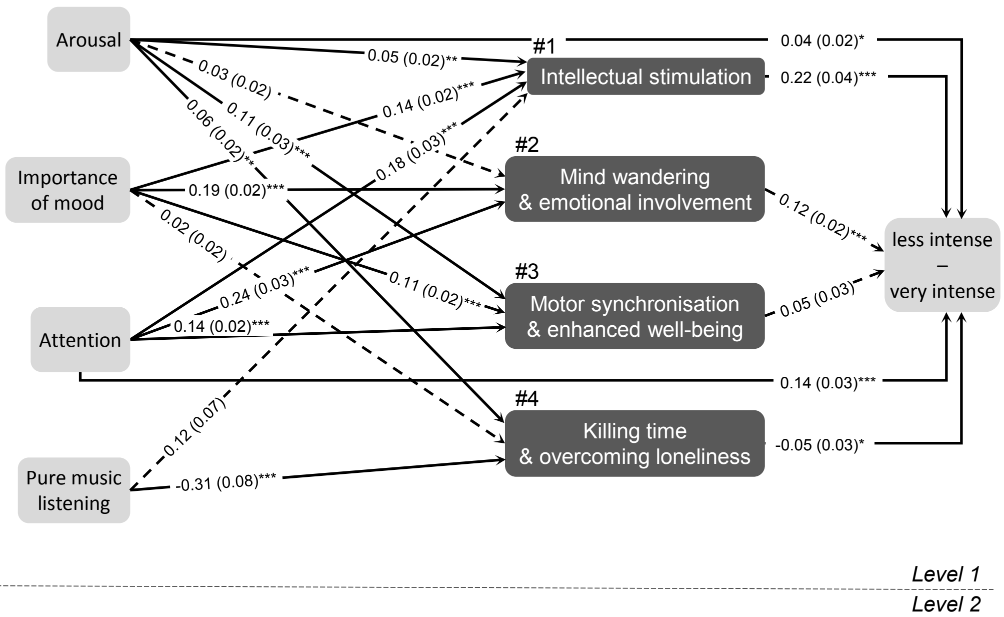

#	Est.	(SE)	p
1:	0.05	(0.05)	.32
2:	0.11	(0.06)	.072

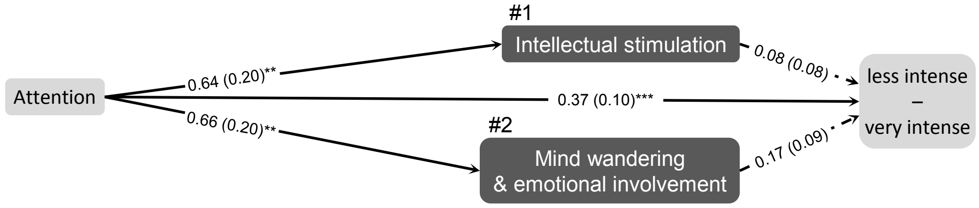

5 References

Greb, F., Schlotz, W., & Steffens, J. (2017). Personal and situational influences on the functions of music listening. *Psychology of Music,* Advance online publication. doi:10.1177/0305735617724883

movisens GmbH. (2015). movisensXS, Version 1.1.0. Karlsruhe: movisens GmbH.
